# Synchronized, Spontaneous, and Oscillatory Detachment of Eukaryotic Cells: A New Tool for Cell Characterization and Identification

**DOI:** 10.1002/advs.202200459

**Published:** 2022-07-03

**Authors:** Derick Yongabi, Mehran Khorshid, Patricia Losada‐Pérez, Soroush Bakhshi Sichani, Stijn Jooken, Wouter Stilman, Florian Theßeling, Tobie Martens, Toon Van Thillo, Kevin Verstrepen, Peter Dedecker, Pieter Vanden Berghe, Minne Paul Lettinga, Carmen Bartic, Peter Lieberzeit, Michael J. Schöning, Ronald Thoelen, Marc Fransen, Michael Wübbenhorst, Patrick Wagner

**Affiliations:** ^1^ Laboratory for Soft Matter and Biophysics Department of Physics and Astronomy KU Leuven Celestijnenlaan 200 D Leuven B‐3001 Belgium; ^2^ Faculté des Sciences Experimental Soft Matter and Thermal Physics (EST) Université Libre de Bruxelles Boulevard du Triomphe ACC.2 Brussels B‐1050 Belgium; ^3^ Laboratory for Systems Biology VIB Center for Microbiology Department of Microbial and Molecular Systems KU Leuven Gaston Geenslaan 1 Heverlee B‐3001 Belgium; ^4^ Laboratory for Enteric Neuroscience (LENS) Department of Chronic Diseases Metabolism and Ageing KU Leuven Herestraat 49 Leuven B‐3000 Belgium; ^5^ Biochemistry Molecular and Structural Biology KU Leuven Celestijnenlaan 200 G Leuven B‐3001 Belgium; ^6^ Biomacromolecular Systems and Processes (IBI‐4) Research Center Jülich GmbH Leo‐Brandt‐Straße D‐52425 Jülich Germany; ^7^ Faculty of Chemistry Department of Physical Chemistry University of Vienna Währinger, Straße 38 Vienna A‐1090 Austria; ^8^ Institute of Nano‐ and Biotechnologies INB Aachen University of Applied Sciences Heinrich‐Mußmann‐Straße 1 D‐52428 Jülich Germany; ^9^ Institute for Materials Research Hasselt University Wetenschapspark 1 Diepenbeek B‐3590 Belgium; ^10^ Laboratory of Peroxisome Biology and Intracellular Communication Department of Cellular and Molecular Medicine KU Leuven Herestraat 49 Leuven B‐3000 Belgium

**Keywords:** cancer therapy, cell characterization, cell detection, glycolytic oscillations, heat‐transfer method, metabolic activity, spontaneous cell detachment

## Abstract

Despite the importance of cell characterization and identification for diagnostic and therapeutic applications, developing fast and label‐free methods without (bio)‐chemical markers or surface‐engineered receptors remains challenging. Here, we exploit the natural cellular response to mild thermal stimuli and propose a label‐ and receptor‐free method for fast and facile cell characterization. Cell suspensions in a dedicated sensor are exposed to a temperature gradient, which stimulates synchronized and spontaneous cell‐detachment with sharply defined time‐patterns, a phenomenon unknown from literature. These patterns depend on metabolic activity (controlled through temperature, nutrients, and drugs) and provide a library of cell‐type‐specific indicators, allowing to distinguish several yeast strains as well as cancer cells. Under specific conditions, synchronized glycolytic‐type oscillations are observed during detachment of mammalian and yeast‐cell ensembles, providing additional cell‐specific signatures. These findings suggest potential applications for cell viability analysis and for assessing the collective response of cancer cells to drugs.

## Graphical abstract

A new method for characterizing eukaryotic cells is described. The approach is based on the observation that under a temperature gradient, eukaryotic cells display spontaneous and collective detachment from a heated solid–liquid interface. This results in an increase in the temperature of the liquid after a sharply defined time, which is temperature dependent and cell‐type specific.

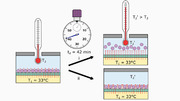

## 1 Introduction

Cell characterization and identification are crucial for medical diagnostic and therapeutic applications. The most common cell detection techniques utilize DNA microarrays, microscopy, flow cytometry, fluorescence‐ and magnetic‐activated cell sorting.^[^ ^1^, ^2^, ^3^ ^]^ While these techniques are powerful, they require lab‐bound equipment operated by trained staff. In addition, functional surface coatings and/or fluorescence labeling are needed, which is expensive, time‐consuming, and the latter potentially changes the intrinsic cell properties.^[^ ^4^, ^5^ ^]^ Efforts toward label‐free methods have employed physical descriptors such as cell size,^[^ ^6^ ^]^ density,^[^ ^1^ ^]^ and dielectric properties.^[^ ^7^, ^8^ ^]^ Unfortunately, these properties tend to vary even within the same cell type.^[^ ^2^ ^]^ Other approaches for cell characterization employ a variety of optical biosensing principles to detect specific biomarkers on cells: This topic is reviewed by Tjandra *et al*.^[^ ^9^ ^]^ Furthermore, relevant information on cells can be retrieved from studying the dynamics of characteristic cellular activities, such as cell adhesion, detachment, and differentiation. Suitable techniques for monitoring these processes in real time include, for instance, quartz crystal microbalances as well as optoelectronic devices that are sensitive to refractive index changes at the solid–liquid interface.^[^ ^1^, ^10^ ^]^ These processes, which are often monitored under uniform temperature conditions are slow, occurring at timescales of several hours, which makes cell characterization along this route less time‐efficient.

Therefore, an unanswered question is how to develop fast, reliable, and label‐free methods for cell characterization and identification that do not require (bio)‐chemical markers or receptors. To this end, opportunities may lie in the knowledge of how cells respond to various stimuli and the resulting morphological and motility processes that occur either at the whole‐cell level, e.g., cell detachment, migration, oscillations,^[^ ^11^, ^12^, ^13^ ^]^ or at the intracellular level, such as the spatiotemporal changes in protein complexes and metabolites.^[^ ^14^, ^15^ ^]^ Thermal stimuli, including local heating, and temperature gradients across cells have a strong potential for inducing morphological and cytoskeletal changes: examples include the formation of membrane extensions,^[^ ^16^ ^]^ redistribution of actomyosin complexes,^[^ ^17^ ^]^ tubulin, and actin polymerization^[^ ^18^, ^19^ ^]^ through symmetry breaking.^[^ ^15^ ^]^ Apart from being able to initiate these cellular processes, temperature gradients can effectively control their spatiotemporal dynamics.^[^ ^16^, ^18^, ^19^ ^]^

**Figure 1:**
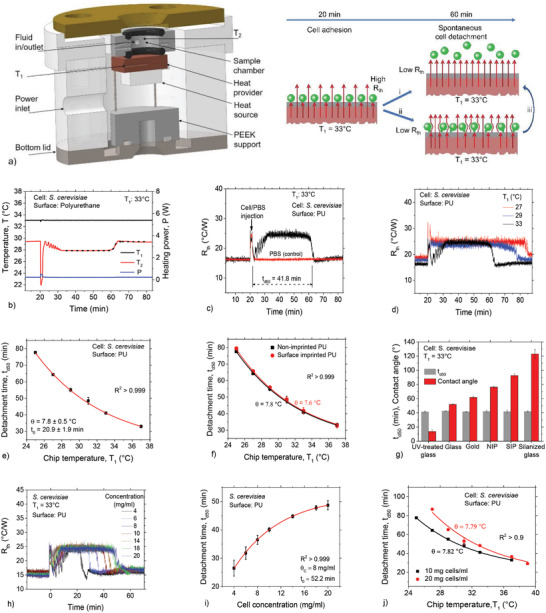
Spontaneous and synchronized yeast detachment. a) Schematic of the HTM platform (left) and detection principle (right). b) Typical measurement depicting the time dependence of the chip temperature, *T*_1_, and the liquid temperature, *T*_2_, in response to cellular interactions at the chip–liquid interface. c) Heat transfer resistance, *R*_th_, as a function of time, showing *R*_th_ changes corresponding to the alteration in *T*_2_, with *t*_d50_ representing the detachment half‐life. No changes occur in the control sample, consisting of PBS without cells. d) Time dependent *R*_th_ for measurements at 27, 29, and 33 °C. e) Plot of *t*_d50_ versus temperature, *T*_1_, for a temperature range of 25–37 °C. The solid line is an exponential decay fit (Equation (1)). f) Comparison of temperature‐dependent *t*_d50_ profiles from polyurethane and imprinted polyurethane surfaces showing similar exponential decay trends, with comparable cell detachment temperature constants, *θ*. g) Correlation between *t*_d50_ measured at 33 °C and water contact angle of various surfaces showing a relatively constant *t*_d50_ for the different surfaces. h) Representative *R*_th_ plots as a function of time at 33 °C for various cell concentrations (C), showing an increase in spontaneous detachment time with C. i) *t*_d50_ as function of cell concentration (*T*_1_ = 33 °C), with *θ* = 7.95 mg mL^−1^ determined from Equation (1). j) Cell detachment time, *t*_d50_ as a function of temperature for 10 and 20 mg mL^−1^, displaying higher *t*_d50_ for the latter, but with comparable *θ* values. Data points in all plots represent at least three measurements and the errors are the standard deviations.

In this work, we propose a label‐ and receptor‐free method for characterizing cells in a fast and facile way by exploiting how cells respond to thermal stimuli. Cells are exposed to temperature gradients in a low‐cost thermal sensor platform: the heat transfer method, HTM^[^ ^20^ ^]^ (**Figure** **1**a; Experimental Section). The temperature gradient induces collective and spontaneous cell‐detachment with sharply defined patterns, which serve as indicators for cell identification, allowing to distinguish noninvasively between closely related yeast strains and human cancer cells.

To gain insights into the spontaneous and synchronized cell detachment phenomenon, we characterized its correlation with cell metabolism by i) probing its dependence on various conditions of temperature, nutrients, and drug dosing, and ii) using a resazurin assay to establish a one‐to‐one correlation between the kinetics of spontaneous detachment and metabolic activity. These studies provide additional cell‐specific detachment signatures, including synchronized and sustained oscillations of large ensembles of cancer and yeast cells. The oscillations are an interesting finding because these spatiotemporal patterns form the basic mechanisms of adaptive behavior in living systems,^[^ ^21^, ^22^, ^23^, ^24^, ^25^ ^]^ including adaptive processes of cancer cells toward chemo‐ and radioresistance.^[^ ^26^, ^27^, ^28^ ^]^ The high sensitivity of spontaneous cell detachment and oscillatory signatures to metabolic activity suggests that, apart from being a cell characterization platform, the proposed approach might be useful for studying the collective response of cells, such as cancer cells to drugs, toward developing more efficient therapies.

## 2 Results

### 2.1 Time‐Dependent Spontaneous Cell Detachment

To characterize cell response to temperature gradients using HTM, we employed a tuneable heat source of power, *P*, which heats the substrate chip from its backside to a predefined constant temperature, *T*_1_. The temperature of the liquid, *T*_2_, is monitored simultaneously (see the Experimental Section). Figure 1a (left) shows a schematic illustration of the HTM device. Regarding the detection principle, adhered cells at the substrate–liquid interface lead to a decrease in *T*_2_, corresponding to an increase in the heat transfer resistance, *R*_th_ [*R*_th_ = (*T*_1_ *–T*_2_)/*P*]. The reverse holds for cell detachment (see Figure 1a, right).

First, we used Dr. Oetker yeast (*Saccharomyces cerevisiae*) as model cells and extended the study to two additional yeast strains (*S. cerevisiae* S288C and *S. pastorianus* W34/70) and two human cancer cell lines: MCF‐7 (breast cancer), and HeLa (cervical cancer). Yeast cells (Dr. Oetker) were provided as dry aggregates and, for simplicity, we report the concentrations in “dry cell weight” per ml of PBS buffer.

Figure 1b compares the liquid temperature, *T*_2_, and the preset chip temperature, *T*_1_, as a function of time, the chip being polyurethane‐coated aluminum. As shown, *T*_1_ (and *P*) remains constant at 33.0 °C throughout the measurement, while *T*_2_ decreases from a stable 29.5 to 27.9 °C after injecting 10 mg mL^−1^ cell suspension. After 41.8 min, *T*_2_ spontaneously recovers to its preinjection value without an external influence. Figure 1c shows the corresponding *R*_th_ changes as a function of time. We hypothesize that the sudden recovery of the interface resistance indicates a spontaneous collective cell detachment from the polymer surface (scenario i, in Figure 1a right).

An alternative hypothesis to full cell detachment that might also lead to an enhanced heat flow through the chip–liquid interface relates to morphological changes on the cells themselves. To this end, the well‐established formation of polarization membrane blebs in response to a temperature gradient, as described in Oyama *et al*. is consistent with this view (scenario ii, Figure 1a right). Finally, since temperature gradient‐induced blebs grow as a function of time, a third scenario can be considered, in which blebs continually extend until cells eventually detach (scenario iii). An assessment of the blebbing hypothesis using blebbistatin as a bleb inhibitor is discussed in Section 2.10. For simplicity therefore, further reference to “detachment” in this paper includes all scenarios for which loss of contact between the substrate chip and the cell occurs, thus ranging from minimally partial detachment due to morphological changes to full loss of contact.

The recovered interface resistance remains constant over many hours (Figure S1a–c, Supporting Information). Based on more than ten repeated experiments, we confirm that this effect happens after a sharply defined time. In addition, correlative HTM‐microscopy measurements on fluorescent *S. cerevisiae* revealed a fluorescence decrease coincident with the drop in *T*_2_ (Figure S1d,e, Supporting Information). Additional imaging experiments were performed on cancer cells (HeLa) and will be discussed in Section 2.12. For convenience, we define the characteristic cell detachment time (or detachment half‐life), *t*_d50_, as the time span between the end of cell injection and the moment when *T*_2_ (or *R*_th_) has recovered by 50% to its value prior to cell injection (Figure 1c). For this, the raw data were analyzed with a sigmoid function as described in the Experimental Section.

### 2.2 Temperature Gradient Controls Spontaneous Cell Detachment

The effect of temperature, and thus temperature gradient (see Figure S1f, Supporting Information, for a correlation) on spontaneous cell detachment was explored by repeating the experiments and analysis in Figure 1b,c at different chip temperatures. Figure 1d displays typical *R*_th_(*t*) plots for three temperatures, *T*_1_ = 27, 29, and 33 °C. The *R*_th_ recovery time, *t*_d50_ becomes systematically longer with decreasing chip temperature (Figure 1e) according to Equation (1)

(1) $$t_{d50}=t_{0}+A\cdot \exp\left(-T_{1}/\theta \right)$$

where *t*_0_ is the horizontal asymptote, *A* is the amplitude, *T*_1_ is the chip temperature in °C, and *θ* is a scaling temperature. Both *t*_0_ and *θ* are constants, and in the following sections, we will show that they depend on the cell type. For the case of Dr. Oetker yeast, their numerical values are *t*_0_ = 20.8 ± 1.8 min and *θ* = 7.8 ± 0.5 °C. In Figure S1g of the Supporting Information, we show that another representation is also possible: *t*_d50_ ∝ 1/*T*_1_.

### 2.3 Gold Does not Always Matter

Now, we show that the substrate material has no significant influence on the detachment half‐life, *t*_d50_. For a direct comparison, we chose polyurethane (PU) coatings that were surface‐imprinted with yeast cells using soft lithography (see the Experimental Section and Figure S1h, Supporting Information). The imprinted PU surface is rougher, contains transferred functional groups and has a higher hydrophobicity, but their *t*_d50_ values are practically identical for all temperatures (Figure 1f). This similarity also holds for the temperature scaling constant, *θ* (Equation (1)), which is 7.8 ± 0.5 °C for nonimprinted and 7.6 ± 0.6 °C for imprinted PU. Similarly, for multiple surfaces of different surface hydrophobicity, including glass, UV‐treated glass, gold, and highly hydrophobic glass (see the Experimental Section for surface preparation), the *t*_d50_ values at *T*_1_ = 33 °C do not show visible differences (Figure 1g). We note that differences were observed in the dynamics of the cell adsorption phase (adsorption level and rate) when compared between the different surfaces, as expected from literature.

### 2.4 Influence of Convection

Toward understanding the physical forces involved in spontaneous detachment, we employed a simulation study (see the Experimental Section ) to evaluate the influence of the convective heat flow in the measuring chamber on cell lift‐off. The simulation result is a stationary single roll convection pattern with data from a 200 µm mesh. Figure S2 of the Supporting Information displays the results of the temperature profiles (upper panels), velocity, and streamlines (middle panels) as well as shear rate (lower panel) in the center of the flow cell for *T*_1_ = 37 °C. Analysis at a lower temperature, *T*_1_ = 30 °C gave similar results. In conclusion, the convective forces are by far not strong enough to cause cell lift‐off.

### 2.5 Effect of Cell Concentration

The influence of cell concentration on *t*_d50_ is displayed in Figure 1h (*R*_th_ vs time) and Figure 1i (*t*_d50_ vs concentration) for a concentration range of 4 to 20 mg mL^−1^. The *R*_th_ recovery time, *t*_d50_, increases exponentially toward a saturation value with increasing concentration. While the absolute *t*_d50_ values depend on the concentration, it is interesting to note that the scaling of *t*_d50_ with respect to the chip temperature is universal: for instance, *θ* ≈ 8 °C for data acquired with 10 and 20 mg mL^−1^ yeast concentrations (Figure 1j). Note that Figure 1h presents only representative raw *R*_th_ data for each concentration without a baseline correction. For a perspective of the dependence of *R*_th_ on cell concentration, see Figure S1i of the Supporting Information.

### 2.6 Effect of an Energy Source: Sucrose

**Figure 2:**
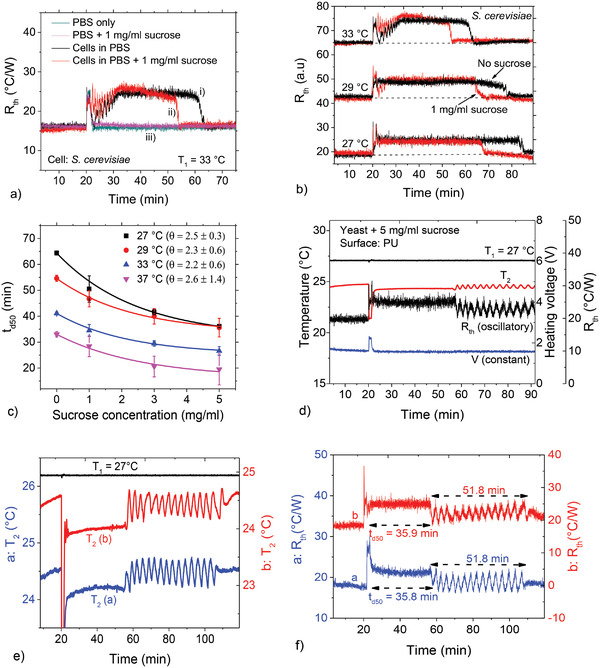
Effect of sucrose on spontaneous yeast detachment and collective oscillations. a) Time‐dependent *R*_th_ plots comparing yeast cell detachment in pure PBS and in 1 mg mL^−1^ sucrose–PBS solution at 33 °C. The cell detachment time is shorter for the measurement involving 1 mg mL^−1^ sucrose. Separate reference measurements in pure PBS and in a 1 mg mL^−1^ sucrose–PBS suspension show no changes in *R*_th_. b) *R*_th_ plots comparing cell detachment for cells in 1 mg sucrose suspension at different temperatures. Data for 29 and 33 °C displaced along the vertical axis for easier visualization. c) Spontaneous cell detachment time, *t*_d50_, as a function of sucrose concentration, showing an exponential decrease in detachment time with increasing sucrose concentration at all temperatures. d) The combined effects of sucrose and temperature reveals a state of sustained oscillatory behavior (*T*_1_ = 27 °C, 5 mg mL^−1^ sucrose) with a sharply defined oscillation starting time, oscillation lifetime as well as the number and period of the oscillations. Oscillations can be unequivocally attributed to cells as confirmed from the constant values of the heating temperature, *T*_1_ and heating voltage, *V*: Low *R*_th_ corresponds to detached cells, while high *R*_th_ means that cells are again adsorbed, thus efficiently blocking thermal transfer as illustrated in Figure 1a. Eventually, the *T*_2_ and *R*_th_ values indicate that the cells stay detached from the chip surface. e,f) Oscillatory time‐dependent *T*_2_ (e) and *R*_th_ (f) responses from independent measurements depicting reproducibility in oscillatory starting time, life span, and number of oscillations.

To probe the mechanisms of cell detachment from a metabolism perspective, we studied the influence of sucrose on the detachment kinetics. We performed measurements with *S. cerevisiae* suspensions (10 mg mL^−1^) containing various sucrose concentrations. As an example, **Figure** **2**a shows *R*_th_ as a function of time for i) cells in pure PBS, ii) cells in PBS containing 1 mg mL^−1^ sucrose, and iii) reference samples lacking cells. While all measurements are performed at *T*_1_ = 33.0 °C, the *R*_th_ recovery time for ii) is 9 min shorter than for i). For other chip temperatures (e.g., 27.0 and 29.0 °C, see Figure 2b), the low dose of 1 mg mL^−1^ sucrose in PBS also results in a clear *t*_d50_ shortening. Figure 2c summarizes the data for four concentrations, *C* = 0.0, 1.0, 3.0, and 5.0 mg mL^−1^ sucrose. At each temperature with its characteristic *t*_d50_ value in pure PBS (*C* = 0.0 mg mL^−1^), the *t*_d50_ reduction follows an exponential trend.

### 2.7 Oscillatory Detachment and Adhesion: A Cell‐Based Metronome

Here, we show that under specific conditions of temperature and sucrose concentration, spontaneous detachment is accompanied by sustained oscillations. Figure 2d shows a typical *R*_th_ time profile for *T*_1_ = 27.0 °C and 5 mg mL^−1^ sucrose: while *T*_1_ and the heater voltage, *V*, are constant, *T*_2_ (and thus *R*_th_) displays periodic and sustained oscillations. Figure 2e,f shows two additional independent measurements under the same conditions. On average, for four tests, the oscillations start 36.0 ± 0.4 min after the end of cell injection and persist for 52 ± 0 min with a period of 3.5 ± 0.6 min. Keeping in mind that *T*_1_ and *V* do not change measurably during this time, but *T*_2_ does, these periodic oscillations must therefore occur at the level of the cells at the solid–liquid interface.

### 2.8 Cell Metabolic Activity Drives Spontaneous Cell Detachment

**Figure 3:**
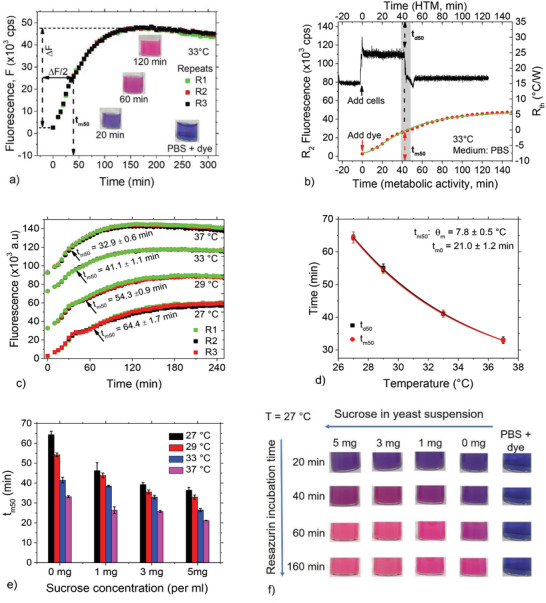
Cell metabolic activity drives spontaneous cell detachment. a) Time‐dependent resorufin fluorescence for three measurements reflecting yeast cell metabolic activity at 33 °C. In all cases, the starting time corresponds to the moment of incubation of the cells in resazurin. Inset: images from cuvettes, showing the time‐dependent color change of resazurin after 20, 60, and 120 min incubation of cells in resazurin. b) Comparison of HTM and metabolic activity fluorescence showing a matching between *t*_d50_ and *t*_m50_. c) Temperature‐dependent resorufin fluorescence for three repeated measurements per temperature and the corresponding *t*_m50_ values. Data for 29–37 °C displaced along the vertical axis for easier visualization. d) Plot of cell metabolic activity half‐life, *t*_m50_ as a function of medium temperature, which perfectly reproduces HTM cell detachment half‐life, *t*_d50_ values and scaling constants, *θ* and *t*_0_ according to Equation (1). e) *t*_m50_ as a function of sucrose concentration. For all plots in (d) and (e), data points are averages of at least three independent measurements and the error bars are the standard deviations. f) Fluorescence images acquired from cuvettes at different time points for yeast suspension as a function of sucrose concentrations at 27 °C as an example, displaying an increase in resorufin fluorescence (pink) with time and sucrose concentrations. PBS + dye (resazurin) without cells was used as a control sample.

We further studied the link between spontaneous cell detachment and cellular metabolism by employing a kinetic study of cell metabolic activity (see the Experimenal Section). Metabolically active cells (10 mg mL^−1^ *S. cerevisiae*) convert resazurin (blue) to resorufin (pink), thus resorufin fluorescence is a measure of cell metabolic activity.^[^ ^29^ ^]^ **Figure** **3**a displays resorufin fluorescence as a function of time from three independent measurements over a period of 5 h at 33 °C. The inserted images show the corresponding color change of the cell suspension. Each plot depicts a nonlinear metabolic behavior, which saturates after ≈2 h.

We define the metabolic activity half‐life, *t*_m50_ as the time to achieve 50% of cell metabolic activity saturation (i.e., Δ*F*/2 in Figure 3a). Based on multiple measurements at 33 °C, *t*_m50_ is 41.5 ± 1.4 min, which coincides with the *t*_d50_ value of 41.1 ± 0.6 min determined by HTM at the same temperature and cell concentration (Figure 1d). Figure 3b depicts the *t*_m50_/*t*_d50_ matching with data from both platforms. Figure 3c shows three repeated measurements at four different temperatures, 27, 29, 33, and 37 °C, displaying clear differences in their *t*_m50_ values. Interestingly, the *t*_m50_ at all temperatures agrees perfectly with the cell detachment *t*_d50_ values (Figure 3d). In addition, *t*_m50_ also follows the exponential scaling of Equation (1). The corresponding *θ* and *t*_0_ are 7.8 ± 0.8 °C and 21.0 ± 1.2 min, in full agreement with the HTM (see Figure 1e).

Further insight into the metabolic argument is provided by a kinetic study of cell metabolic activity at various sucrose concentrations. As shown in Figure 3e, the presence of sucrose shortens *t*_m50_ in the same concentration‐dependent manner described for the HTM data in Figure 2c. Correlative fluorescence images are displayed in Figure 3f for measurements at 27 °C. The fluorescence plots as a function of temperature and sucrose concentrations are provided in Figure S3a–d of the Supporting Information. The absolute *t*_d50_ and *t*_m50_ values are comparable for all temperatures and sucrose concentrations (Figure S3e, Supporting Information).

### 2.9 Effect of Dimethyl Sulfoxide (DMSO) on Spontaneous Cell Detachment

**Figure 4:**
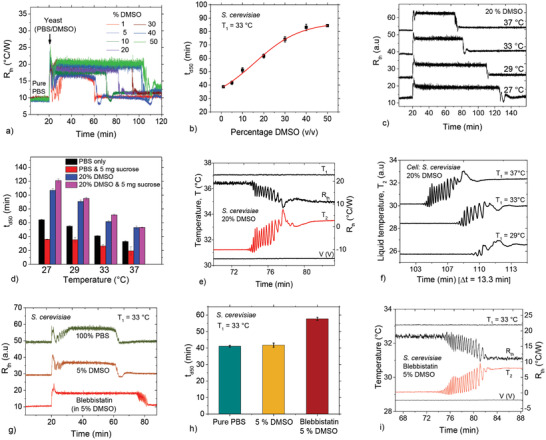
Effect of DMSO and blebbistatin on spontaneous *S. cerevisiae* detachment. a) Time‐dependent *R*_th_ displaying spontaneous cell detachment for various DMSO concentrations: detachment time lengthens with increasing percentage DMSO. Cells were incubated in various DMSO concentrations for 60 min prior to measurements. b) *t*_d50_ as a function of DMSO concentration, showing an increase in *t*_d50_ with increasing DMSO concentration, consistent with a dose–response trend depicted by the solid line. For up to 5% DMSO, *t*_d50_ increases only moderately. c) Real‐time *R*_th_ plots for *S. cerevisiae* cells in 20% DMSO for various temperatures. Data for 29–37 °C are shifted along the *R*_th_ axis for easier comparison. d) Comparison of *t*_d50_ for *S. cerevisiae* in various media at different temperatures. e) HTM parameters displaying oscillations in liquid temperature, *T*_2_, and *R*_th_ for cells in 20% DMSO and *T*_1_ = 37 °C. The heater voltage and *T*_1_ are stable, meaning that the oscillations are intrinsically cellular. f) *T*_2_ as function of time during spontaneous detachment, showing a decrease in the number of oscillations with decreasing chip temperature, *T*_1_. g) *R*_th_ plots as a function of time showing a strong effect of blebbistatin (1 mm, in 5% DMSO/95% PBS) on cell detachment: measurements in blebbistatin show a considerable delay in *R*_th_ recovery in comparison with reference measurements (pure PBS and 5% DSMO). h) Comparison of the effect of blebbistatin on cell detachment time for an average of three measurements, with the errors being the standard deviations. i) Oscillations in *T*_2_ and *R*_th_ reflective of blebbistatin‐induced cellular oscillations.

To further study the mechanisms of spontaneous cell detachment as a metabolically driven process, we used DMSO, which negatively impacts cell viability at high concentrations in a dose‐dependent way.^[^ ^30^, ^31^ ^]^ **Figure** **4**a displays the time dependent *R*_th_ response of *S. cerevisiae* (10 mg cells mL^−1^) for different DMSO concentrations at 33 °C (vol% for all DMSO concentrations). The detachment time, *t*_d50_ increases systematically from 39.0 ± 0.4 min in 1% DMSO to 84.0 ± 0.4 min for 50% DMSO (Figure 4b). With increasing volume fraction of DMSO, *t*_d50_ follows a dose–response trend, depicted by the solid line in Figure 4b. The delaying effect of DMSO on collective cell detachment occurs at all chip temperatures, *T*_1_ (Figure 4c). Interestingly, DMSO (20%) completely cancels the accelerating effect of sucrose (Figure 4d).

The detachment phase in the presence of DSMO shows oscillatory features, which are visible in *T*_2_ and *R*_th_ (Figure 4e). Decreasing *T*_1_ reduces the oscillation frequency as well as the number of discernible oscillations (Figure 4f). Regarding the effect of DMSO on cell metabolic activity, Figure S4a–f of the Supporting Information confirms that unlike the cells in pure PBS, cells in 20% DMSO display markedly lower metabolic activity at all temperatures (27–37 °C).

To ascertain that the sustained oscillations at 27 °C in sucrose are related to cell metabolism (Figure 2d–f), we analyzed the behavior of the *R*_th_ responses for cells measured under the same sucrose and temperature conditions, but with lower metabolic activity (1 h incubation in 20% DMSO). As shown in Figure S4g,h of the Supporting Information, the detachment time for cells in DMSO is much longer and without sustained oscillations. As further proof, when Baker's yeast cells, stored at room temperature for 3 years (thus expired) were measured at 27 °C, neither detachment nor oscillatory signatures were observed: The results under sucrose and non‐sucrose conditions are similar (data not shown).

### 2.10 Blebbistatin Retards Spontaneous Cell Detachment

Next, we performed measurements on cells incubated with blebbistatin, which is a highly selective inhibitor for nonmuscle myosin II.^[^ ^32^ ^]^ As depicted in Figure 4g,h, blebbistatin delays spontaneous detachment, with *t*_d50_ longer for blebbistatin‐treated cells by ≈16 min. Also, for blebbistatin‐treated cells, the detachment process includes numerous high‐frequency oscillations (Figure 4i), which are similar in timescales to those of DMSO (<10 min) but differ markedly when compared with the 52 min oscillations observed with 5 mg mL^−1^ sucrose (Figure 2).

### 2.11 Spontaneous Cell Detachment Kinetics Is Cell‐Type Specific

**Figure 5:**
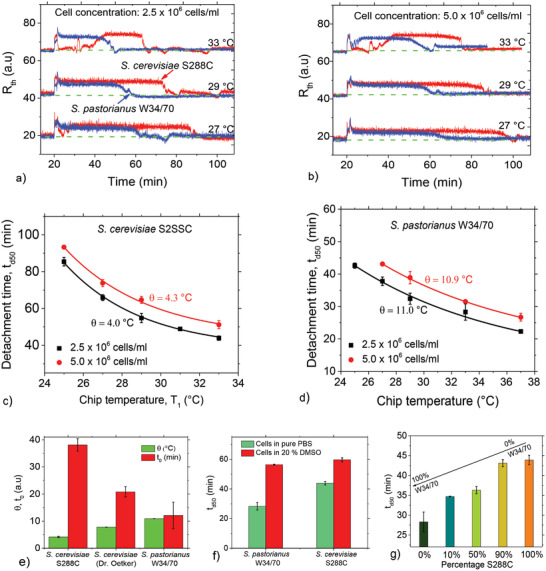
Spontaneous cell detachment kinetics is cell‐type specific. a,b) Time‐dependent *R*_th_ showing significantly longer cell detachment times for *S. cerevisiae* S288C in comparison with *S. pastorianus* W34/70 at various temperatures for two cell concentrations, 2.5 × 10^6^ and 5 × 10^6^ cells mL^−1^, respectively. For both strains, cell detachment time shortens as the temperature increases. In (a) and (b), the curves for different temperatures, *T*_1_ are displaced vertically along the *R*_th_ axis for clarity. c) Temperature‐dependent *t*_d50_ for 2.5 × 10^6^ and 5 × 10^6^ cells mL^−1^ of *S. cerevisiae* S288C, displaying exponential trends with comparable *θ* values. d) Similar data for *S. pastorianus* equally showing higher *t*_d50_ values for the higher concentration, but with similar *θ* values. e) Comparison of the average temperature constant, *θ*, by yeast strain showing marked differences between all three cells (Dr. Oetker yeast included). The asymptotical value of *t*_d50_, i.e., *t*_0_ also differs by cell type. f) Comparison of *S. pastorianus* and *S. cerevisiae* S288C adhesion and spontaneous detachment in pure PBS and in 20% DMSO, showing a lengthening of *t*_d50_ for both cell types in 20% DMSO compared to pure PBS for an average of three measurements. g) Spontaneous detachment of cell mixtures: cell detachment time, *t*_d50_, as a function of relative concentrations of *S. cerevisiae* S288C and *S. pastorianus*.

To explore the cell‐type‐dependence of spontaneous detachment, we used two additional yeast strains, *S. cerevisiae* S288C and *S. pastorianus* W34/70. The results are displayed in **Figure** **5**a for a concentration of 2.5 × 10^6^ cells mL^−1^ and various chip temperatures, *T*_1_ (27, 29, and 33 °C). The *R*_th_ recovery shows that these cells spontaneously detach as well. For all *T*_1_, the detachment time, *t*_d50_, for *S. cerevisiae* S288C is considerably longer than for *S. pastorianus* irrespective of the cell concentration (Figure 5a,b). For both cell types, *t*_d50_ increases with decreasing *T*_1_.

The *t*_d50_ plots as a function of *T*_1_ are shown in Figure 5c,d for *S. cerevisiae* S288C and *S. pastorianus*, respectively for 2.5 × 10^6^ and 5 × 10^6^ cells mL^−1^. As an example, for *S. cerevisiae* S288C at *T*_1_ = 33 °C, detachment times vary from 44.0 ± 1.2 min for 2.5 × 10^6^ cells mL^−1^ to 51.2 ± 2.2 min for 5 × 10^6^ cells mL^−1^. The corresponding *t*_d50_ values for *S. pastorianus* under the same conditions are 28.3 ± 2.5 min and 31.5 ± 0.3 min for the lower and higher concentrations, respectively. This difference is remarkable, given the similarity of both cells, and it holds for each *T*_1_ and cell concentration. As depicted in Figure 5c,d, *t*_d50_ as a function of *T*_1_ shows that the data are consistent with the function in Equation (1) (*R* ^2^ > 0.99 in all cases). For each cell type, the scaling temperature constant, *θ*, is the same for both concentrations: for a concentration of 2.5 × 10^6^ cells mL^−1^, we measured 4.0 ± 0.5 °C for *S. cerevisiae* S288C and 11.0 ± 3.1 °C for *S. pastorianus*. The corresponding values for 5 × 10^6^ cells mL^−1^, are 4.3 ± 0.9 °C for *S. cerevisiae* S288C and 10.9 ± 2.1 °C for *S. pastorianus*, hence the same for the two concentrations, but clearly different between both yeast strains.

Another cell‐type dependent fit parameter is the horizontal asymptote *t*_0_ of Equation (1). Figure 5e shows a comparison of *t*_0_ and *θ* of the yeast strains (2.5 × 10^6^ cells mL^−1^) with data for Dr. Oetker *S. cerevisiae* included. Both parameters differ clearly when comparing the three strains. While *θ* does not depend on cell concentration or surface material, *t*_0_ depends weakly on the concentration. In the case of *S. cerevisiae* S288C, *t*_0_ ≈ 38 min for 2.5 × 10^6^ cells mL^−1^ and increases to ≈44 min for 5.0 × 10^6^ cells mL^−1^. With *S. pastorianus*, *t_0_* increases from 12 to 16 min when comparing the same two concentrations. Hence, the concentration‐related variability is much smaller than the intrinsic difference of 26 min between the two strains, *S. cerevisiae* S288C and *S. pastorianus* (Figure 5e). Finally, we showed that the influence of DMSO on spontaneous detachment and the oscillatory behavior observed for Dr. Oetker yeast also holds for *S. pastorianus* and *S. cerevisiae* S28CC (Figure S5a–c, Supporting Information).

Regarding mixtures of *S. cerevisiae* S288 and *S. pastorianus* at a given temperature (e.g., *T*_1_ = 33 °C) and total cell concentration (2.5 × 10^6^ cells mL^−1^), the short detachment time for *S. pastorianus* increases as the relative amount of *S. cerevisiae* S288C increases (Figure 5g; Figure S6, Supporting Information). For 50% relative concentration of both strains, the *t*_d50_ value is at the midpoint (≈36 min) of the values for pure *S. pastorianus* (≈28 min) and pure *S. cerevisiae* S288C (≈44 min).

### 2.12 Spontaneous Detachment of Human Cancer Cell Lines

**Figure 6:**
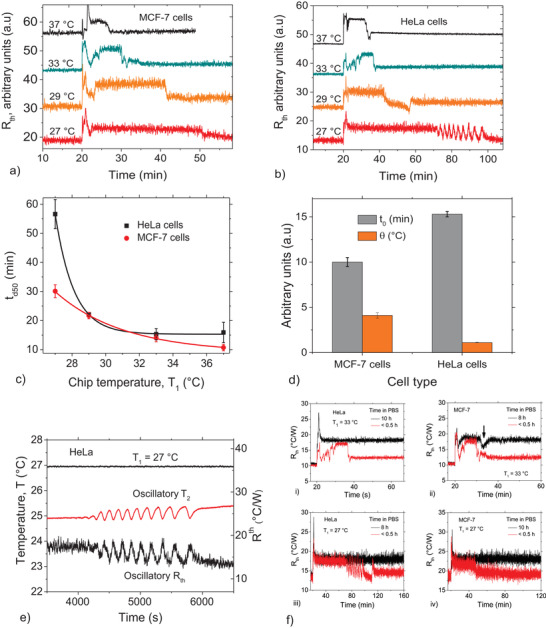
Spontaneous detachment of human cancer cell lines. a,b) *R*_th_ as a function of time, representing cell detachment for the human cell lines, MCF‐7 (a) and HeLa (b) for representative measurements at different temperatures. Curves are displaced vertically for easier comparison. c) Comparison of temperature‐dependent *t*_d50_ plots for MCF‐7 and HeLa cells displaying an exponential decay trend for both. d) Scaling parameters, *θ* and *t*_0_, compared between MCF‐7 and HeLa cells. All errors in (c) and (d) are standard deviations from at least three measurements. e) Time‐dependent oscillations of the interfacial resistance, *R*_th_, and liquid temperature, *T*_2_, for HeLa cells measured at 27 °C. f) HeLa and MCF‐7 measurements at 33 and 27 °C on cells incubated in PBS for 10 h compared to cells measured less than 30 min after exchange of culture medium with PBS: cells incubated in PBS for 10 h remained attached, probably due to ATP deficiency, and suggests that detachment is associated with cell metabolism. Also, only cells that had been quickly switched from culture medium to PBS and measured immediately displayed sustained oscillations, confirming that these oscillations are dissipative structures that arise as the cells adapt to the changing nutrient conditions.

To show that spontaneous detachment also occurs in human cell lines, we explored the effect on breast‐cancer (MCF‐7) and cervical cancer (HeLa) cell lines. The cell concentrations were uniformly ≈1.0 × 10^6^ cells mL^−1^ and we used the same measurement protocols as described for the yeast strains above. For MCF‐7 and HeLa cells, **Figure** **6**a,b shows the *R*_th_(*t*) profiles for different chip temperatures, *T*_1_, confirming that these human cells also display spontaneous detachment. Figure 6c summarizes the *t*_d50_ data for both cell lines. As a general trend, the absolute *t*_d50_ values are higher for HeLa cells in comparison with MCF‐7 cells. The exponential scaling of Equation (1) also applies for *t*_d50_ as a function of *T*_1_ (Figure 6c). The scaling parameters are *t*_0_ = 10.0 ± 0.5 min for MCF‐7 and 15.3 ± 0.3 min for HeLa, while the corresponding *θ* values are 4.1 ± 0.3 °C (MCF‐7) and 1.1 °C (HeLa) (Figure 6d). Therefore, in addition to the absolute detachment time, *t*_d50_, the scaling parameters, *θ* and *t*_0_, differ considerably for the cancer cell types studied.

We also observed oscillations during cancer cell detachment (Figure 6b). Sustained oscillations show up at *T*_1_ = 27 °C for HeLa cells (Figure 6e; Figure S7a, Supporting Information, for details), and last for 25.2 ± 0.1 min with a period of 3.4 ± 0.9 min. For MCF‐7, oscillations were also observed, but at a lower temperature, *T*_1_ = 26 °C (see Figure S7b, Supporting Information).

To assess the influence of cell metabolism (and ATP availability) on the spontaneous detachment of cancer cells, we performed additional measurements on cells incubated in nutrient‐free 1× PBS for a minimum 8 h prior to HTM measurements. Figure 6f shows that for measurements at 33 °C (panels i and ii) and 27 °C (iii and iv), the MCF‐7 and HeLa cells do not detach under these nutrient deprivation conditions for the entire measurement time.

**Figure 7:**
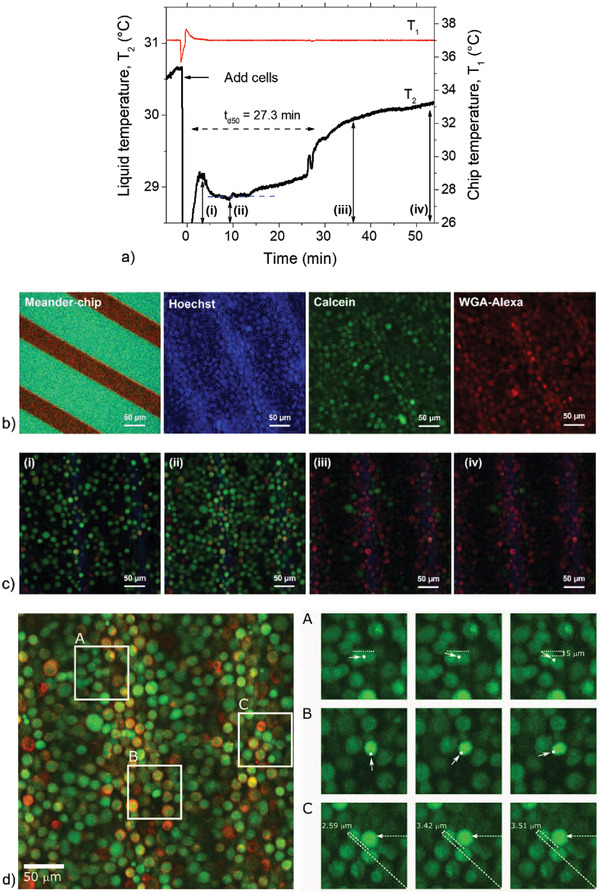
Correlative HTM‐microscopy monitoring of HeLa cells. a) Comparison of Meander‐chip temperature, *T*_1_ and liquid temperature, *T*_2_, from an HTM monitoring experiment of HeLa cells in the microscope chamber. b) Left: optical image of meander on glass. The three right images in (b) show fluorescence of Hoechst 33342, Calcein‐AM, and WGA‐Alexa Fluor 594 from cells attached to the meander chip. c) Images of cells on the chip surface acquired during the HTM measurement shown in (a) at time points corresponding to (i), (ii), (iii), (iv) and depicting rounded cells. d) Left: image acquired during the measurement in (a) at *t* = 16 min (during *T*_2_ recovery, dashed line as reference). Right upper panel in (d): zoomed‐in of region (A) displaying images of cells during the detachment phase for three timeframes corresponding to 16, 16.7, and 17.3 min and depicting cell rotational and translational motion of a reference cell indicated with a white dot. The right middle panel (zoom‐in of (B)) shows a cell also displaying motion at the moment of *T*_2_ recovery: 20.8, 21.5, and 22 min in the HTM signals (panel a). Right lower panel: two cells displaying both rotation and translational motion.

For a better understanding of the state of individual cells and spatiotemporal behavior before, during and after spontaneous detachment events, we performed live imaging on cancer cells (HeLa) due to their large sizes compared to the yeast trains. Live imaging experiments were performed on cell suspensions subjected to a temperature gradient as described in the Experimental Section (Optical Verification of Cell Detachment section). **Figure** **7**a shows the liquid temperature, *T*_2_ and the chip temperature, *T*_1_ as a function of time. While *T*_1_ stays constant throughout, *T*_2_ recovers, starting from 14 min after cell injection. The average *t*_d50_ is 23.2 ± 6 min (for repeated measurements), which is comparable to the data in Figure 6c, considering the differences in technical details of the flow chamber adapted for microscopy observation and the effect of temperature drift caused by photon interactions during imaging (see Figure S8, Supporting Information, for details).

To facilitate visualization, cells were labeled with WGA‐Alexa Fluor 594 (plasma membrane stain), and Hoechst 33342 (nuclei stain) as well as Calcein‐AM to ascertain that the cells were viable after treatment. Figure 7b shows a microscope image of the meander‐chip (heater) exposed to PBS in the HTM sensing compartment as well as the fluorescence from the three labels from the same image of adsorbed cells on the chip. The Calcein‐AM fluorescence indicates that cells remained viable following treatment with the fluorescence dyes. Calcein‐AM bleaches with time due to photon exposure. In terms of real time live imaging, Figure 7c shows images of cells adsorbed on the chip surface acquired during the HTM monitoring experiment shown in a) at time points corresponding to (i), (ii), (iii), and (iv). During the entire measurement, cells remain rounded and uniformly distributed on the surface, meaning that cell spreading did not occur during the time span of the experiment. The *T*_2_ signal decrease from the moment of cell loading to a stable level (as indicated by the dashed line) is clearly correlated with the surface coverage from image (i) to image (ii). We observe from the live video (data not shown) that cells stream in and remain mobile for up to 5 min as the surface coverage increases, before becoming stationary on the surface. Cells remain immobile until 14 min after injection. The stationary state corresponds to the low *T*_2_, consequently high *R*_th_. After the stationary phase, one can observe the motion of individual cells, which are both rotatory and translational, going along with a recovery of the *T*_2_ signal. Examples of these cellular movements are presented in Figure 7d, which corresponds to 16 min of the HTM time in panel a). This timeframe falls within the regime of *T*_2_ recovery and is also consistent with the repeated measurements (see Figure S8a, Supporting Information). The right upper panel in d) is a zoom‐in of region A) displaying images of cells during the detachment phase for three timeframes, corresponding to 16.0, 16.7, and 17.3 min in a). The timescale of the HTM data is adjusted to begin at 0 min from the moment of cell loading: real time = 10 min in PBS + 1.2 min cell injection. The highlighted cell (see white dot to guide the eye) is clearly seen exhibiting a clockwise rotatory motion as well as a translational displacement of 5 µm. The right middle panel (zoom‐in of region B) also shows a cell displaying similar movements at the moment of *T*_2_ recovery in HTM. The images were acquired from three frames corresponding to 20.8, 21.5, and 22 min in the HTM signals (panel a). Finally, the images in the right lower panel of d) depict two cells in rotation and translational motion starting at 25 min in the HTM profile and which also coincides perfectly with the *t*_d50_ value of 27.3 min indicated in a). The movie also reveals multiple cellular movements spanning the entire region of the *T*_2_ recovery phase. These movements give an indication of cells in a state of detachment: one would expect rotation of loosely attached cells, as in the case of a cell that becomes tethered on a bleb, or deviation in the path of a detached cell due to repulsive interactions from neighboring cells. Also, a cell in a detaching state under local convection due to enhanced heat flow might create rotations, as seen by the antisense rotation of neighboring cells in the movie (data not shown).

We note here limitations that make visualization of cellular movements along the *z*‐axis, as well as detailed morphological changes in this direction, hard to observe: the objective of the microscope used has a working distance of 570 µm and data acquisition is achieved from the bottom of the HTM sensing chamber, which is 500–600 µm thick, thus also explaining the low resolution of images, in addition to photon scattering within the glass‐gold meander‐glass chip assembly of the HTM sensor. Also, multiple photon excitations cause photobleaching of the fluorescence dyes with time. Finally, the spontaneous cell detachment effect is a temperature‐dependent phenomenon, while photon excitation causes significant changes in the temperature of the entire system (microscope chamber, HTM sensing compartment), feedback effects, and drifts in the heater voltage, all of which affect the experimental conditions, thus cellular behavior (see Figure S8, Supporting Information). Therefore, further studies are needed with a more dedicated imaging system to decern movement along the *z*‐axis.

## 3 Discussion

To the best of our knowledge, the real‐time observation of synchronized and spontaneous cell detachment has not been reported before. The exponential scaling of *t*_d50_ with temperature (e.g., Figure 1e) is a first indication that the effect depends on metabolic activity.^[^ ^17^, ^33^ ^]^ A fundamental temperature‐dependent metabolic process that is strongly linked to eukaryotic cell adhesion and detachment is cell polarization.^[^ ^15^, ^34^ ^]^ Cell polarization is the basic mechanism that drives cytoskeletal reorganization and leads to spatiotemporal events, which control single‐ and collective‐cell behaviors.^[^ ^35^ ^]^ Unpolarized cells are symmetrical in terms of the distribution of cytoplasmic components (e.g., actin filaments, myosin II motor proteins), and their associated signaling proteins.^[^ ^12^, ^13^, ^15^ ^]^ In polarized cells, this symmetry is broken through mechanisms that involve positive and negative feedback processes.^[^ ^35^, ^36^, ^37^ ^]^ Symmetry breaking in cells is highly sensitive to various gradient stimuli, including chemical, electrical, and thermal gradients.^[^ ^15^, ^16^, ^17^, ^18^, ^19^ ^]^ Therefore, we hypothesize that the spontaneous cell detachment observed is due to temperature gradient‐induced cell polarization. Various polarization‐dependent processes can modulate cell adhesion and detachment, including i) focal adhesion formation and disassembly,^[^ ^34^, ^38^ ^]^ ii) cell migration,^[^ ^39^, ^40^, ^41^ ^]^ and iii) formation of membrane extensions.^[^ ^16^ ^]^

With regards to the cellular temperature response mechanisms, all eukaryotic cells, including yeast and mammalian cells have built‐in mechanisms for responding to temperature gradients and heat shocks through transient receptor potential ion channels.^[^ ^42^ ^]^ By means of these cellular thermosensors, small temperature gradients are capable of stimulating eukaryotic cells to break symmetry and polarize,^[^ ^18^, ^19^ ^]^ including yeast^[^ ^13^ ^]^ and mammalian cells.^[^ ^16^ ^]^ The formation of membrane blebs in response to a temperature gradient as described by Oyama *et al*.^[16^ ^]^ is consistent with the delayed cell detachment observed for blebbistatin‐treated cells because this drug inhibits the formation of membrane extensions (Figure 4g,h). This suggests that polarization‐induced blebs may play a role in the spontaneous detachment mechanism. High‐resolution imaging of cells at the substrate–liquid interface would be useful for a conclusive visual picture.

### 3.1 Evidence for Metabolic Activity as a Driver of Collective Cell Detachment

The sucrose‐yeast measurements confirm that cell metabolism is a driving force for spontaneous cell detachment: The shorter *t*_d50_ values with increasing sucrose concentration mean that the process is energy‐dependent. Furthermore, the kinetic study of resazurin reduction by *S. cerevisiae* as a function of temperature and sucrose concentration provides additional proof. The one‐to‐one association between the spontaneous detachment half‐life and cell metabolic activity half‐life (see Figure 3b,d) is an interesting finding with important consequences: spontaneous detachment monitoring can serve as a real‐time, noninvasive tool for assessing cell metabolic activity. The inhibitory effect of DMSO on the spontaneous detachment of all yeast strains is further support of the metabolic argument, considering the negative effects of DMSO on cell metabolic activity.^[^ ^30^, ^31^ ^]^

### 3.2 Spontaneous Cell Detachment Reveals Cell‐Specific Discriminators

The results on the three yeast strains demonstrate that HTM‐based spontaneous cell detachment monitoring generates cell‐type specific fingerprints that can be used for selective cell identification. Furthermore, the measurements with mixtures of *S. cerevisiae* S288C and *S. pastorianus* W34/70 (Figure 6) indicate that spontaneous cell detachment kinetics is sensitive to the relative amount of each constituent cell type, which supports the applicability of the strategy for cell analysis in more complex systems.

Regarding cancer cells, the differences in the absolute detachment times *t*_d50_, (Figure 6a–c) and the scaling parameters, *θ* and *t*_0_ (Figure 6d), compared between MCF‐7 and HeLa cells reveal that these parameters can also be useful for characterizing and identifying cancer cells. The exponential temperature‐dependence of *t*_d50_, together with the results in Figure 6f is equally indicative of a cell metabolic activity dependence.

### 3.3 Oscillatory Features during Yeast and Cancer Cell Detachment

Our measurements also revealed pronounced oscillations during spontaneous cell detachment. Oscillations arise from negative feedback and are crucial to the functioning of biological and cellular processes.^[^ ^27^, ^36^, ^43^ ^]^ For instance, bud formation, and migration in yeast,^[^ ^13^, ^43^, ^44^ ^]^ as well as mammalian cell detachment and migration^[^ ^39^, ^45^ ^]^ have been associated with oscillations of periods in the seconds–few minutes range. While the periods of our observed oscillations fall within this timescale, we measure large cell ensembles, and their dependence on sucrose, temperature, and drugs (DMSO and blebbistatin) confirms that they are metabolically driven.^[^ ^42^ ^]^

### 3.4 Do Oscillations of Yeast and Cancer Cells Reflect Glycolytic Oscillations?

Glycolytic oscillations are periodic fluctuations in the concentrations of the metabolites of glycolysis.^[^ ^14^, ^46^, ^47^ ^]^ They are mostly studied by fluorescence monitoring,^[^ ^27^, ^48^, ^49^ ^]^ or molecular modeling.^[^ ^14^, ^25^, ^50^ ^]^ While experiments have mostly focused on intracellular observations following the addition of glucose and enhancing chemicals (e.g., cyanide), a few studies have reported on synchronized and sustained oscillations in yeast cell populations, lasting up to ≈40 min.^[^ ^46^, ^48^, ^49^ ^]^ This agrees with the 52 min sustained oscillations we measure in the presence of high sucrose concentration in Figure 2d–f. *S. cerevisiae* is well‐known to enhance its glycolytic oscillations at high glucose concentrations, and this should hold same for sucrose. We note that glycolytic oscillations have been associated with fluctuations in metabolic heat flux,^[^ ^51^ ^]^ but our results do not reflect such heat fluxes. In the case of metabolic heat flux oscillations, one would expect a decrease in the temperature of the liquid at the end of the oscillations, because the heat produced would be quickly dissipated to the environment. In our case (e.g., Figure 2d,e), *T*_2_ remains high and constant after the oscillatory phase.

MCF‐7 and HeLa cells also displayed synchronized glycolytic‐type oscillations under different conditions (Figure 6; Figure S7, Supporting Information). While individual cancer cells are known to display glycolytic oscillations,^[^ ^52^ ^]^ to date, no studies have reported on direct and clear oscillations of large ensembles of cancer cells in real‐time. Recently, by using fluorescence‐microscopy, Amemiya *et al*. reported glycolytic oscillations in individual HeLa cells with periods between 0.5 and 2 min, depending on the number of cells analyzed. We note that in our case, collective oscillations are observed for cell populations in the order of 10^6^ cells mL^−1^ in real‐time, and noninvasively. Oscillations have important pharmacological applications. For instance, studies indicate that cancer cells with higher glycolytic activity are more malignant, more prone to resisting immune T cell therapy, and display higher chemo‐ and radioresistance.^[^ ^26^, ^47^ ^]^

## 4 Conclusion

In summary, the results from this work clearly show that our strategy can be used to effectively characterize and potentially identify eukaryotic cells, including human cancer cells. The high sensitivity of the reported effect to cell metabolic activity presents important opportunities for, e.g., pharmacological effect studies in drug screening and toxicity assessment. The oscillatory features may offer additional fine‐tuned information on cells. Furthermore, the proposed method is fast: depending on the chip temperature and the concentration of nutrients or drugs, the characteristic detachment time can be as small as 10–20 min for cancer cells and 23–45 min for yeast strains. The concept is also facile to implement since it requires not more than a PID controlled‐heating resistor and two thermometers, in our case miniature thermocouples. The chip material itself plays only an insignificant role and there are no bioreceptors or surface patterning involved. Other benefits include the noninvasive character of the method, which uses a very mild temperature gradient as a stimulus to trigger the cell response. The detachment times are sharply defined, highly reproducible, and statistically solid as they are retrieved in real time from large cell ensembles. As a brief outlook, it is the purpose to study the reported effect on a broader range of eukaryotic cells, especially cancer cell lines, and to upgrade the platform toward measuring in a broader temperature range, by including, for instance, an option for active cooling of the chip.

## 5 Experimental Section

### Cell Preparation

Dry aggregates of commercial *S. cerevisiae* (Dr. Oetker, Bielefeld, Germany) were rehydrated by dispersing in 1× PBS buffer (pH 7.4, 137 mM NaCl, 2.7 mm KCl, 8 mm Na_2_HPO_4_, and 2 mM KH_2_PO_4_) at room temperature and gently agitated for 1 min by vortexing at 500 rpm. Different concentrations were prepared by dispersing cells at various mass/volume ratios. Therefore, all concentrations are expressed in dry cell weight per milliliter (mL) PBS. From the *R*_th_ read‐out perspective, a high cell concentration range, from 4 to 20 mg mL^−1^ was used to ensure that the *R*_th_ signals were always large enough to allow for a decent visualization of the spontaneous detachment regimes. This results in a smaller trade‐off being a slight difference in the absolute values of the *R*_th_ as a function of concentration. To study the effect of cell metabolism on detachment, sucrose (table sugar) was used as an energy source, since sucrose uptake is a direct proof of cell metabolic activity.^[^ ^53^ ^]^ Different amounts of sugar, in mg mL^−1^, were dissolved in the cell suspensions to vary the sucrose concentrations. *S. cerevisiae* was used because of its fully documented genetic sequence^[^ ^54^ ^]^ and its wide use as a model for understanding eukaryotic cell biochemistry in response to external cues.^[^ ^35^, ^37^, ^55^ ^]^

The yeasts strains, *S. cerevisiae* S288C and *S. pastorianus* W34/70, which are used in brewing lager beers^[^ ^56^ ^]^ were cultivated by inoculating precultures at 30 °C in a growth medium containing yeast extract (1% w/v), glucose (2% w/v), and peptone (2% w/v), followed by shaking for 24 h at 200 rpm. Cells were harvested by centrifuging for 5 min at 2000 × *g*. The cells were washed twice in abundant 1× PBS to remove residues of the culture medium and resuspended in 1× PBS. A TC‐20 Automated Cell Counter (Bio‐Rad, Hercules, USA) was used to count the cells to obtain specific concentrations. The concentrations used for measurements were comparable to the concentration range of Dr. Oetker yeast.

MCF‐7 cells (ATCC HTB‐22) and HeLa cells (ATCC CCL‐2) were kindly provided by Dr. Mieke Verstuyf (KU Leuven, Belgium) and Dr. Patrizia Agostinis (KU Leuven, Belgium), respectively. The cells were cultured at 37 °C in a humidified 5% CO_2_ incubator in alpha MEM Eagle (Lonza, BE12‐169F; for MCF‐7) or DMEM: F12 (Lonza, BE12‐719F; for HeLa) medium supplemented with 10% (v/v) fetal bovine serum (Biowest, S181B), 2 mm ultraglutamine‐1 (Lonza, BE17‐605E/U1), and 0.2% (v/v) Mycozap (Lonza, VZA‐2012). The culture medium was refreshed every 2–3 days, and the cells were subcultured before confluency by trypsinization with 1× trypsin/EDTA (Lonza, BE02‐007E) at a 1:5 ratio. The cell number was determined using an Assistent Bürker counting chamber (Karl Hecht KG, Germany). To assess the effect of cell metabolism on collective cancer cell detachment, cells were starved in PBS for 10 h, prior to HTM measurements. These cell lines were used because of their medical relevance as versatile models for the development of novel diagnostic applications in cancer screening as well as for cytostatic drugs performance analysis.^[^ ^57^, ^58^ ^]^

### Treatments with DMSO and Blebbistatin

To explore the mechanisms of collective cell detachment further, DMSO and blebbistatin were used. DMSO [(CH₃)₂SO] is a pharmacological solvent that makes cell membranes permeable for small drug molecules and is widely used for cryopreservation (10 vol% concentrations). At concentrations above 10%, DMSO is cytotoxic in a dose‐dependent manner, making it suitable for probing the effect of cell metabolic activity on spontaneous detachment.^[^ ^30^ ^]^ The cytotoxicity of DMSO results from many processes, including dehydration and oxidative stress, inhibition of ATP hydrolysis, and mitochondrial ATP production.^[^ ^31^, ^59^, ^60^ ^]^ DMSO (purity 99.5%) was purchased from Sigma‐Aldrich N.V. (Overijse, Belgium). To test the effect of DMSO on cell adhesion and detachment, cells were first dispersed in PBS before being supplement with DMSO. Cell suspensions (constant cell concentration) with different DMSO concentrations (v/v) were prepared by varying the DMSO/PBS ratio. Measurements were performed on cells in various percentage DMSO, ranging from 1% to 50% (v/v). Myosin II ATPase was inhibited by 1 mM (‐)‐blebbistatin (purity 99.37), purchased from Apexbio Technology LLC (Houston, USA). Blebbistatin was first dissolved in DMSO before being added to 1× PBS solution to yield a final solution consisting of 5% DMSO (95% PBS) and 1 mm blebbistatin. This DMSO dose is low enough to avoid cytotoxicity, but sufficient to support the cellular uptake of blebbistatin. Prior to the adhesion and detachment monitoring experiments, the cell suspension was kept for 1 h at room temperature to allow for cells to absorb blebbistatin.

### Polymer Synthesis and Coating

PU was synthesized by dissolving 0.122 g of 4,4′ diisocyanatodiphenylmethane, 0.222 g bisphenol A, and 0.025 g phloroglucinol in 500 µL anhydrous tetrahyofuran (THF) under an inert nitrogen atmosphere. All reagents were used as received from Sigma‐Aldrich N.V. (Overijse, Belgium) with a minimal purity of 99.9%. The solution was semicured at 65 °C for 200 min under continuous stirring, and the polymer gel was further diluted in THF in the ratio 1:5 before surface‐coating.

### Surface Preparation

Polyurethane layers were prepared on aluminum chips (1 cm × 1 cm, Brico N.V., Korbeek‐Lo, Belgium). A thin polymer gel layer of about 1.2 µm thickness was deposited on the chips by spin‐coating at 2000 rpm for 60 s with an acceleration of 1000 rpm s^−1^. After deposition, the polymer was fully cured at 65 °C for 12 h under nitrogen atmosphere.

To compare cell adhesion and detachment between plain polyurethane layers and polyurethane layers with altered chemical and topographical features, surface imprinting was employed for the latter. Surface‐imprinted polyurethane layers were synthesized as described in previous work.^[61^ ^–^ ^64^ ^]^

Hydrophilic glass substrates were prepared by exposing microscope cover‐glass chips (Glaswarenfabrik Karl Hecht GmbH & Co, Sondheim, Germany) to UV ozone (UVO‐Cleaner, Jelight Company Inc., CA, USA) for 90 min and cleaned in absolute ethanol and acetone. Hydrophobic glass substrates were prepared by silanization and involved immersing microscope cover glass chips into a 0.5% (v/v) solution of n‐octadecyltrichlorosilane (OTS) in toluene for 1 h and subsequently rinsing three times with water‐free toluene.

For gold‐coated substrates, the gold coatings were prepared by physical vapor deposition at 5 × 10^−5^ Pa. A 20 nm adhesion chromium film was first applied on a silicon chip followed by evaporation of a 100 nm gold layer. Then, the substrates were cleaned by dipping them into a cold piranha solution (H_2_O_2_:H_2_SO_4_ in a 1:3 ratio) for 5 s, followed by rinsing with deionized water. All materials were blow dried with N_2_ after cleaning in various liquids.

### Surface Analysis

SEM images of imprinted and non‐imprinted polymer surfaces were acquired using an FEI Quanta 200F‐scanning electron microscope (FEI, Hillsboro, Oregon, USA) on carbon‐coated samples. Water contact angle measurements were performed on all types of substrates using an optical contact angle measuring system (DataPhysics, OCA 25, Filderstadt, Germany) to evaluate the hydrophobicity of the surfaces. A 5 µL sessile water drop was dispensed onto the chip surfaces at a rate of 5 µL min^−1^ at a room temperature of 18 °C. Measurements were performed on three different specimens for each category of substrates.

### Heat Transfer‐Based Cell Adhesion and Detachment Monitoring

The interaction of the cells with the surfaces at a constant temperature gradient was monitored using the HTM transducer platform.^[^ ^14^, ^65^ ^]^ Figure 1a (left panel) shows a schematic diagram of the HTM device. The measurement area on the substrate chip (1 cm × 1 cm) was defined by an O‐ring acting as a fluid‐tight seal in a 160 µL home‐made Perspex microfluidic cell. The backside of the substrate was mechanically fixed to a copper block, which serves as a heat source, heated by a 20 Ω power resistor (MPH20, Farnell, Belgium). Two automated pump‐driven syringes (ProSense, NE‐500, The Netherlands) were used to exchange the medium (PBS to cell suspension) within the flow cell. Two 500 µm diameter, K‐type thermocouples (TC Direct, The Netherlands) were used to monitor the temperature in the liquid, *T*_2_, 4 mm above the sensor chip surface and the temperature of the heating copper block, *T*_1_. The substrate temperature was maintained at the preset levels using a proportional‐integral‐derivative controller (PID setting 1‐6‐0). The ambient temperature for all measurements was 18.0 ± 0.5 °C. Due to the excellent thermal contact between the chip and the heating copper block, *T*_1_ represents the temperature of the backside of the substrate chip. For linguistic convenience, *T*_1_ is used interchangeably with the chip/substrate temperature.

The interaction of cells with the surfaces was monitored by measuring the time‐dependent temperature, *T*_2_, of the liquid from which the heat transfer resistance (*R*_th_) at the interface between chip and liquid was determined. The time evolution of the *R*_th_ signal was calculated from the temperature difference (*T*_1_–*T*_2_) and the supply power, *P* used to maintain the substrate chip at the set temperature as shown in Equation (2)

(2) $$R_{\mathrm{th}}=\frac{\left(T_{1}-T_{2}\right)}{P}$$

*R*_th_ responds sensitively to changes at the solid–liquid interface down to molecular scales.^[^ ^66^ ^]^ In addition to having a temperature gradient as an intrinsic property, the substrate materials for the proposed HTM measurements are inexpensive and require almost no preparation. Compared to other techniques for studying adhesion and detachment of cell populations based on shear–force assays^[^ ^67^ ^]^ and microgravimetry,^[^ ^68^ ^]^ HTM does not exert mechanical perturbations (hydrodynamic shear and vibrational forces) and provides real‐time monitoring capabilities.

The thermal transport experiments were carried out as follows: First, *T*_1_ was stabilized in PBS buffer for 20 min, after which a cell suspension was injected by the automated pumps at a rate of 2.5 mL min^−1^ for 72 s. Under constant chip temperature and nonflow conditions, the temperature of the liquid, *T*_2_, was monitored as a function of time. Cell adhesion experiments were performed at different constant chip temperatures *T*_1_. The temperature gradient was varied passively by changing the temperature of the heat source, *T*_1_. The heating temperature, *T*_1_ ranged from 25 to 37 °C. This range was chosen because below 25 °C, the temperature gradient is too small for a measurable thermal current, while temperatures above 37 °C may cause cell apoptosis.

A characteristic cell detachment time, *t*_d50_, was defined as the time span between the end of cell injection and the moment when *T*_2_ (or *R*_th_) has recovered by 50% back to its initial value. For better accuracy, the *t*_d50_ values were determined using the sigmoid Boltzmann fit function (Equation (3)) within the OriginPro software package (OriginPro 2016, version b9.3.226, Northampton, MA, USA)

(3) $$Y=A_{2}+\frac{A_{1}-A_{2}}{1+\exp\left(\left(t-t_{1/2}\right)/\Delta t\right)}$$

where *A*_1_ and *A*_2_ are the values of *T*_2_ before and after spontaneous detachment, respectively, Δ*t* is the time constant and *Y* = *T*_2_ (*t*), with *t* being the time. The parameter *t*_1/2_ represents the time during which *Y* is midway between *A*_1_ and *A*_2_. The absolute *t*_1/2_ value characterizes the detachment time of the cell population. Therefore, for absolute *t*_1/2_ calculations, only the time following the end of cell injection was considered. This parameter is referred as the cell detachment half‐life, *t*_d50_, meaning the time for 50% of cell detachment, as a physical interpretation.

### Optical Verification of Cell Detachment

To demonstrate that the cells detach physically from the chip surface at the collective detachment moment, *t*_d50_, a reference experiment was designed, allowing to monitor the chip‐to‐liquid interface from underneath using an inverted microscope. A schematic of the experimental setup is shown in Figure S1d of the Supporting Information. The heating element was composed of an on‐chip meander structure, prepared, and calibrated as described by Cornelis *et al*.^[^ ^69^ ^]^ Fluorescence microscopy data on *S. cerevisiae* were acquired simultaneously using the system specified by Roebroek *et al*.,^[^ ^70^ ^]^ making use of green fluorescence protein excitation and detection channel. A general CRISPR/Cas9 protocol was used to generate constitutively fluorescent S288C *S. cerevisiae*​ cells for correlative measurements.^[^ ^71^ ^]^ Image processing and average intensity trace calculations were performed using the Localizer software package.^[^ ^72^ ^]^

Additional live imaging experiments were also carried out on HeLa cells to visualize the state and behavior of cells during cell adsorption and detachment. To assess cell viability and stain the plasma membrane and nucleus, the harvested cells for live imaging were resuspended in DPBS containing 2 µm Calcein‐AM (Biolegend, 425201), 2 µg mL^−1^ Alexa Fluor 594 wheat germ agglutinin (Thermo Fisher Scientific, W11262), 1 µm Hoechst 33342 (Thermo Fisher Scientific, 62249) and incubated for 30 min in the dark at 37 °C. Thereafter, the cells were pelleted and resuspended in prewarmed cell culture medium for 1 h. Timelapses at 0.1 Hz were recorded simultaneously with HTM monitoring: before, during, and after cell loading for at least 1 h. The HTM sensing compartment was fixated in the microscope chamber and cell loading was achieved by exchanging PBS with a cell suspension using automated syringe pumps as described above (in “Heat Transfer‐Based Cell Adhesion and Detachment Monitoring” section). An LSM 780 confocal laser scanning microscope with ZEN black software (Zeiss) was used. Three channels were recorded in line scan mode and detected with three spectral windows using 2 PMTs and a 32‐channel GaAsP spectral detector. Spectral windows were: i) 397–490 nm for Hoechst 33342 excited with 405 nm laser line, ii) 490–570 nm for Calcein‐AM excited with 488 nm laser line, and iii) 592–751 nm for Alexa Fluor 594 wheat germ agglutinin excited with the 561 nm laser line. To acquire signals through the heater (glass‐meander‐glass assembly) of the HTM‐sensing compartment, an objective with a working distance of 570 µm (Zeiss, 25×, 0.8 NA, water‐immersion) was used. Images were adjusted for brightness and contrast in ImageJ/Fiji.

### Cell Metabolic Activity Measurements

Cell metabolic activity as a function of time was measured using the resazurin cell viability reagent (purity > 99%, Acros Organics, Thermo Fisher Scientific, Geel, Belgium). The working principle is based on the reduction of resazurin (blue) to resorufin (pink) by the respiratory chain of the mitochondrion in live cells. Thus, real‐time resorufin monitoring provides direct information on the kinetics of the metabolic activities of the cell population. Resazurin solution (15 µM) was prepared in autoclave‐sterilized 1× PBS (pH 7.4). Cell suspensions for analysis were prepared in pure 1× PBS as well as in 1× PBS spiked with different sucrose and DMSO concentrations. The cell suspensions were then incubated with resazurin solution (10% v/v) in a six‐well plate. The fluorescence of resorufin was measured from 100 µL aliquots using a Tecan infinite 200PRO microwell plate reader (Tecan Trading AG, Männedorf, Switzerland) at 590 nm wavelength. Each experiment was performed at a preset temperature for at least 4 h in automated (kinetic) mode, with the fluorescence signals measured every 5 min. For each cell suspension sample, fluorescence was also measured from a control solution, with the same liquid composition, but lacking cells.

To quantify the cell metabolic activity, the characteristic time at which the fluorescence intensity is 50% of the value at saturation was used. Since the fluorescence intensity is a measure of cell metabolic activity, this time is denoted as *t*_m50_. While *t*_m50_ can be determined manually from the raw data, the OriginPro‐embedded logistic function shown in Equation (4) was employed for better accuracy

(4) $$Y=A_{2}+\frac{A_{1}-A_{2}}{1+{\left(t/t_{1/2}\right)}^{p}}$$

where *Y* is the resorufin fluorescence as function of time (*t*), *A*_1_ and *A*_2_ are the initial and final values of *Y*, respectively, and *p* is the power. The parameter *t*_1/2_ is the time at which *Y* is midpoint between *A*_1_ and *A*_2_, thus, has the same meaning as in Equation (3). The *t*_1/2_ values therefore correspond to the cell metabolic activity half‐life of the cell population, addressed therefore as *t*_m50_.

### Simulation Study of Convective Heat Flow

To assess the role of convective heat flow within the measuring flow cell (Figure 1) on cell detachment, a simulation study was carried out according to the methods described by Stilman *et al*.,^[^ ^65^ ^]^ and the cell‐lifting shear rate was calculated following the model given by Krishnan and Leighton.^[^ ^73^ ^]^ The convective heat transport through the flow cell is modeled as follows

(5) $$\begin{array}{cc} & \rho C_{p}u\cdot \nabla T-\kappa \nabla^{2}T=0\;\left(\mathrm{energy}\;\mathrm{balance}\right)\\ & \rho \left(u\cdot \nabla \right)u=\nabla \cdot \left[-\mathrm{pI}+\mu \left(\nabla u+{\left(\nabla u\right)}^{T}\right)\right]+\rho g\;\left(\mathrm{momentum}\;\mathrm{balance},\;\mathrm{incompressible}\;\mathrm{flow}\right)\\ & \nabla \cdot \left(\rho u\right)=0\;\left(\mathrm{mass}\;\mathrm{balance}\right) \end{array}$$

With *ρ* [kg m^−^^3^] the density, *C_p_* [J (kg K)^−1^] the specific heat capacity, *T* [K] temperature, *κ* [W (m K)^−1^] the thermal conductivity, *u* [m s^−1^] the velocity, *p* [Pa] the pressure, *μ* [Pa s^−1^] the dynamic viscosity, *g* [N kg^−1^] the gravitational constant, and *I* the unity tensor. A no‐slip boundary condition was assumed on the walls of the fluid cell. Cooling of the sensor occurs by external convection (room temperature), which is defined by an outward heat flux proportional to the temperature difference with the surroundings.^[^ ^65^ ^]^ The nonlinear problem is solved with Comsol's GMRES iterative solver in a fully coupled approach. The mesh convergence is studied for three different sizes of tetrahedral meshes (600, 300, and 200 µm) to determine the shear stress above the sensor chip surface using Richardson extrapolation.

## Conflict of Interest

The authors declare no conflict of interest.

## Author Contributions

D.Y. and P.W. designed the study approach and experiments. P.W. and M.W. obtained funding for the study. D.Y. conducted the experiments, performed data analyses, and wrote the original manuscript with input from all co‐authors and supervision from P.W. M.K., and P.L.‐P. provided input on the HTM study approach and data evaluation. W.S. assisted with HTM measurements. M.F. cultured cancer cell lines (MCF‐7 and HeLa) and assisted with designing the study on these cells. S.J. and C.B. contributed to correlative cell metabolic activity measurements. M.K., S.B.S., T.V.T., P.D., and M.J.S. provided input for fluorescence‐HTM measurements. F.T. and K.V. assisted with yeast cultures, *S. cerevisiae* S288C and *S. pastorianus* W34/70. P.L., M.J.S., and R.T. contributed to the experimental design. M.P.L. contributed to the simulations on the convective forces. P.V.B. and T.M. provided input on live imaging on HeLA cells.

## Supporting information

Supporting Information

## Data Availability

The data that support the findings of this study are available from the corresponding author upon reasonable request.
