# Supplementary material for: Synchronized, Spontaneous, and Oscillatory Detachment of Eukaryotic Cells: A New Tool for Cell Characterization and Identification
Source: Adv Sci (Weinh). 2022 Jul 3;9(24):2200459. doi: 10.1002/advs.202200459 (PMC9403630; doi:10.1002/advs.202200459)
Supplement: Supplementary file 1 — Supporting Information [file ADVS-9-2200459-s001.pdf]

ADVANCED SCIENCE
Open Access

Supporting Information

for *Adv. Sci.*, DOI 10.1002/advs.202200459

Synchronized, Spontaneous, and Oscillatory Detachment of Eukaryotic Cells: A New Tool for Cell Characterization and Identification

Derick Yongabi, Mehran Khorshid, Patricia Losada-Pérez, Soroush Bakhshi Sichani, Stijn Jooken, Wouter Stilman, Florian Theßeling, Tobie Martens, Toon Van Thillo, Kevin Verstrepen, Peter Dedecker, Pieter Vanden Berghe, Minne Paul Lettinga, Carmen Bartic, Peter Lieberzeit, Michael J. Schöning, Ronald Thoelen, Marc Fransen, Michael Wübbenhorst and Patrick Wagner**

Supporting Information

Synchronized, spontaneous and oscillatory detachment of eukaryotic cells – A new tool for cell characterization and identification

Derick Yongabi* [1], Mehran Khorshid [1], Patricia Losada-Pérez [2], Soroush Bakhshi Sichani [1], Stijn Jooken [1], Wouter Stilman [1], Florian Theßeling [3], Tobie Martens [4], Toon Van Thillo [5], Kevin Verstrepen [3], Peter Dedecker [5], Pieter Vanden Berghe [4], Minne Paul Lettinga [1], Carmen Bartic [1], Peter Lieberzeit [6], Michael J. Schöning [7], Ronald Thoelen [8], Marc Fransen [9], Michael Wübbenhorst [1], and Patrick Wagner* [1].

1) KU Leuven, Department of Physics and Astronomy, Laboratory for Soft Matter and Biophysics, Celestijnenlaan 200 D, B-3001 Leuven, Belgium
2) Université Libre de Bruxelles, Faculté des Sciences, Experimental Soft Matter and Thermal Physics (EST), Boulevard du Triomphe ACC.2, B-1050 Brussels, Belgium
3) Laboratory for Systems Biology, KU Leuven VIB Center for Microbiology, Department of Microbial and Molecular Systems, Gaston Geenslaan 1, B-3001 Heverlee, Belgium
4) KU Leuven, Department of Chronic Diseases Metabolism and Ageing, Laboratory for Enteric Neuroscience (LENS), Herestraat 49, B-3000 Leuven, Belgium
5) KU Leuven, Biochemistry, Molecular and Structural Biology, Celestijnenlaan 200 G, B-3001 Leuven, Belgium
6) University of Vienna, Faculty of Chemistry, Department of Physical Chemistry, Währinger Straße 38, A-1090 Vienna, Austria
7) Aachen University of Applied Sciences, Institute of Nano- and Biotechnologies INB, Heinrich-Mußmann-Straße 1, D-52428 Jülich, Germany
8) Hasselt University, Institute for Materials Research, Wetenschapspark 1, B-3590 Diepenbeek, Belgium
9) KU Leuven, Department of Cellular and Molecular Medicine, Laboratory of Peroxisome Biology and Intracellular Communication, Herestraat 49, B-3000 Leuven, Belgium

*Corresponding authors

Dr. Derick Yongabi
Laboratory for Soft Matter and Biophysics ZMB,
Celestijnenlaan 200 D, B-3001, Leuven, Belgium
Email: derick.yongabi@kuleuven.be

Prof. Dr. Patrick Wagner
Laboratory for Soft Matter and Biophysics ZMB,
Celestijnenlaan 200 D, B-3001, Leuven, Belgium
Email: patrickhermann.wagner@kuleuven.be

Supplementary Figures

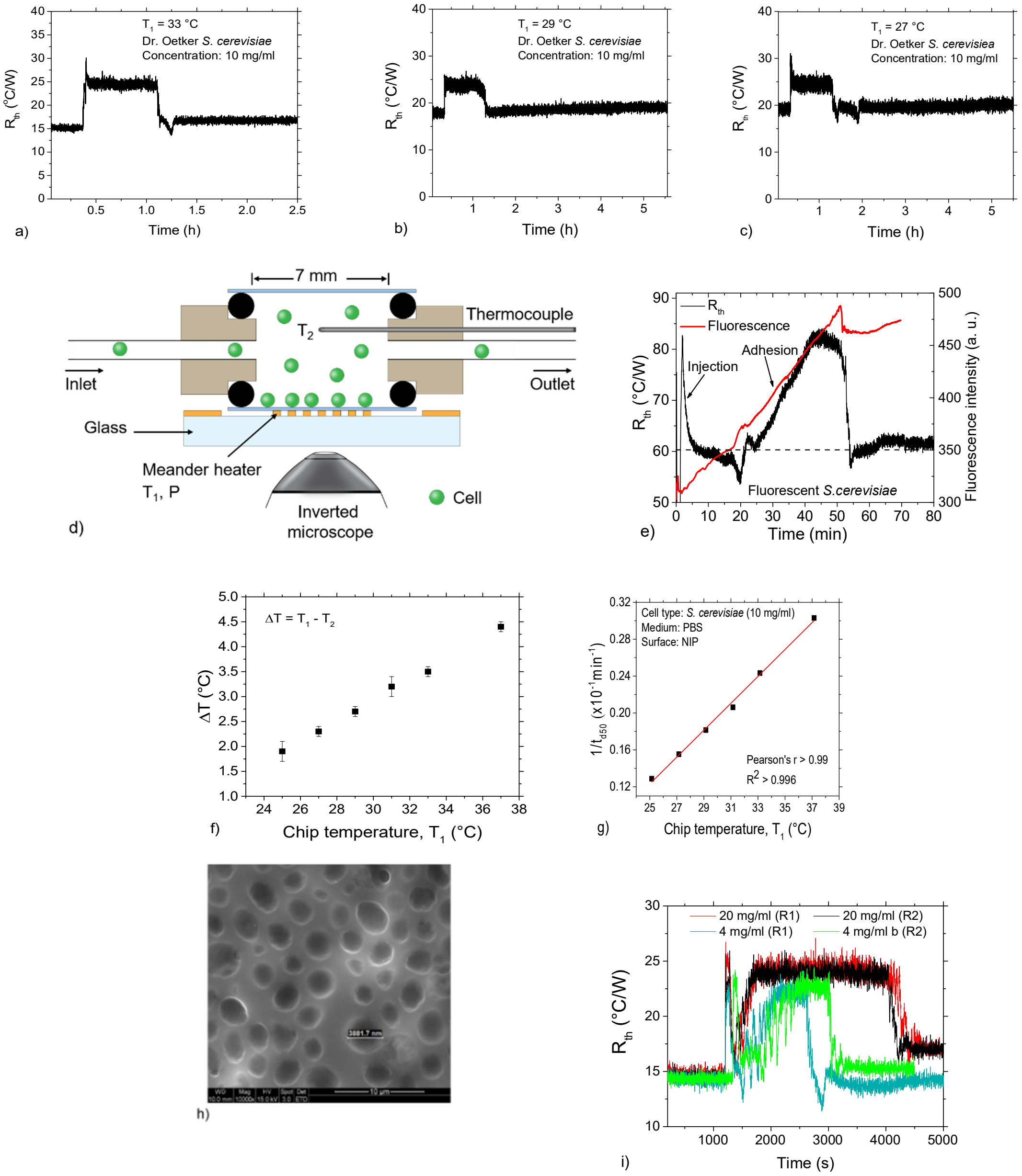

Supplementary Figure S1. Spontaneous and irreversible cell detachment – Correlative fluorescence analysis, temperature, and surface dependence. a-c*, Long-term R_{th} plots as function of time for different temperatures. All data show that the R_{th} recovery due to cell detachment is maintained over long time scales. Set up for simultaneous HTM and microscopy experiment, which allows to monitor the chip-to-liquid interface from underneath using an inverted fluorescence microscope with a meander system as a heater (**d**). The cells were fluorescently labelled yeast cells, expressing the green fluorescent protein (GFP). The outcome of the experiment in which the drop in the thermal resistance signal at $t_{d50} \approx 52$ min coincides with a sudden drop in the fluorescence intensity (**e**). Noteworthy, the fluorescence signal does not drop back to its baseline: This indicates that cells, after detachment, are still in the proximity of the chip surface where they contribute to the intensity since the focus depth of the microscope reaches into the liquid. This reference experiment was performed several times and, while the drop of R_{th} was observed consistently, the decrease in the fluorescence signal could not always be observed sharply, confirming that the upward displacement of cells is limited. The absolute t_{d50} value of 52 min agrees well to data obtained for the laboratory S. cerevisiae S288C at the same concentration of 5×10^6 cells/ml and chip temperature of T_1 = 33 °C in **Figure 5b**. **f**, Linear scaling model: The inverse of t_{d50} displays a linear trend with the chip temperature. **g**, Temperature difference between T_1 and T_2 as a function of chip temperature. We note that the values reported are not the absolute values across single cells, since we measure the gradient macroscopically (see **Methods**). **h**, SEM image of surface-*

imprinted polyurethane displaying surface-imprinted cavities, geometrically matching with yeast cells in shape and size. ***i****,* *Time dependent* R_{th} *responses for repeated measurements at 20 mg/ml and 4 mg/ml yeast concentrations showing a higher* R_{th} *response for the former compared to the latter.*

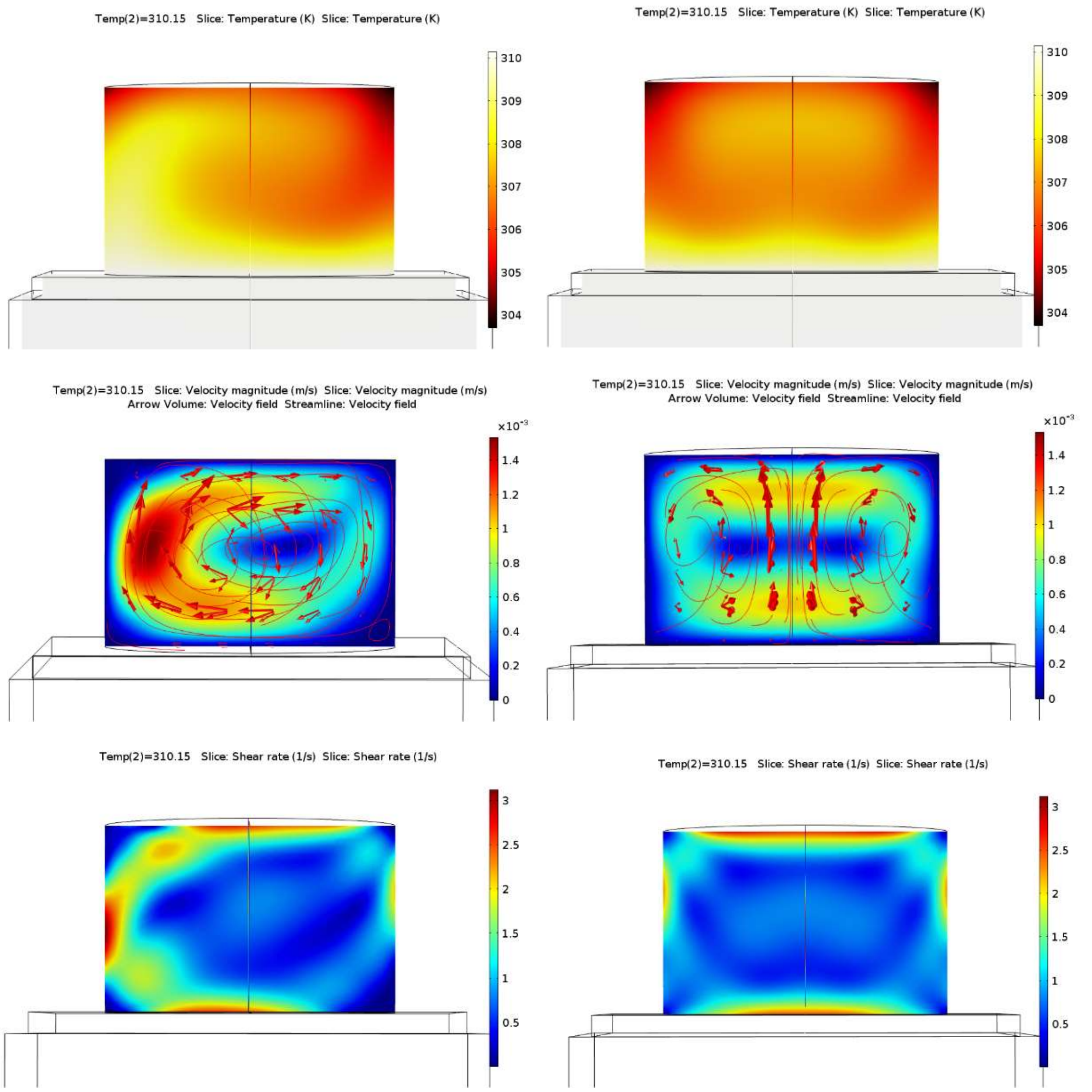

Supplementary Figure S2. Convective flow patterns in the HTM flow cell. Upper panels: *Temperature profile in the centre of the fluid cell (left: yz, right: xz plane) for a temperature of 37°C.* ***Middle panels:*** *Velocity profile and streamlines in the centre of the fluid cell (left: yz, right: xz plane) for a temperature of 37°C.* ***Lower panels:*** *Shear rate in the centre of the fluid cell (left: yz, right: xz plane) for a temperature of 37 °C.*

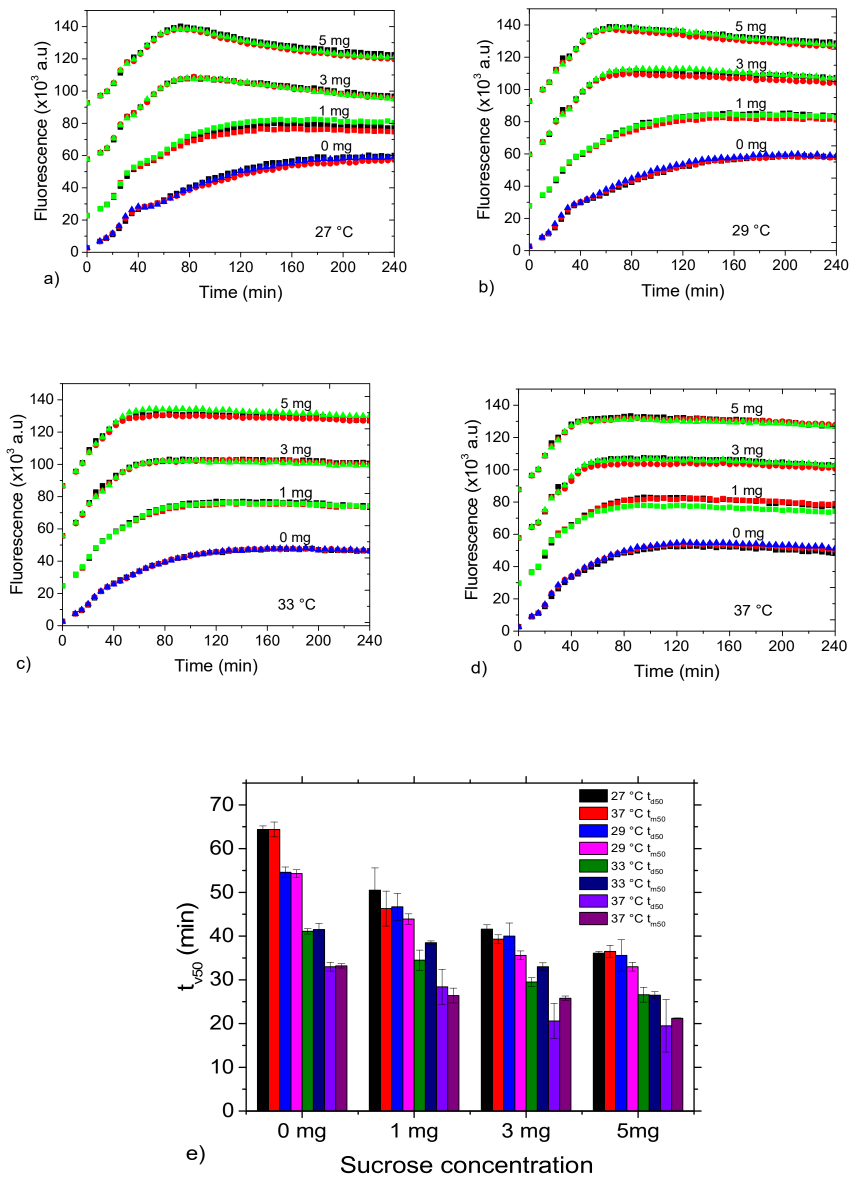

Supplementary Figure S3. Temperature-dependent effect of sucrose on yeast metabolic activity. a-d*, Time-dependent yeast (Dr. Oetker) metabolic activity (resorufin fluorescence) at various temperatures and sucrose concentrations.* ***e****, Comparison of yeast metabolic activity half-life and cell detachment half-life for different sucrose concentrations and temperatures. The absolute* t_{d50} *and* t_{m50} *values are largely comparable for various temperatures and sucrose concentrations; the slight differences displayed in some cases can be attributed to the influence of sucrose on the refractive index of the medium.*

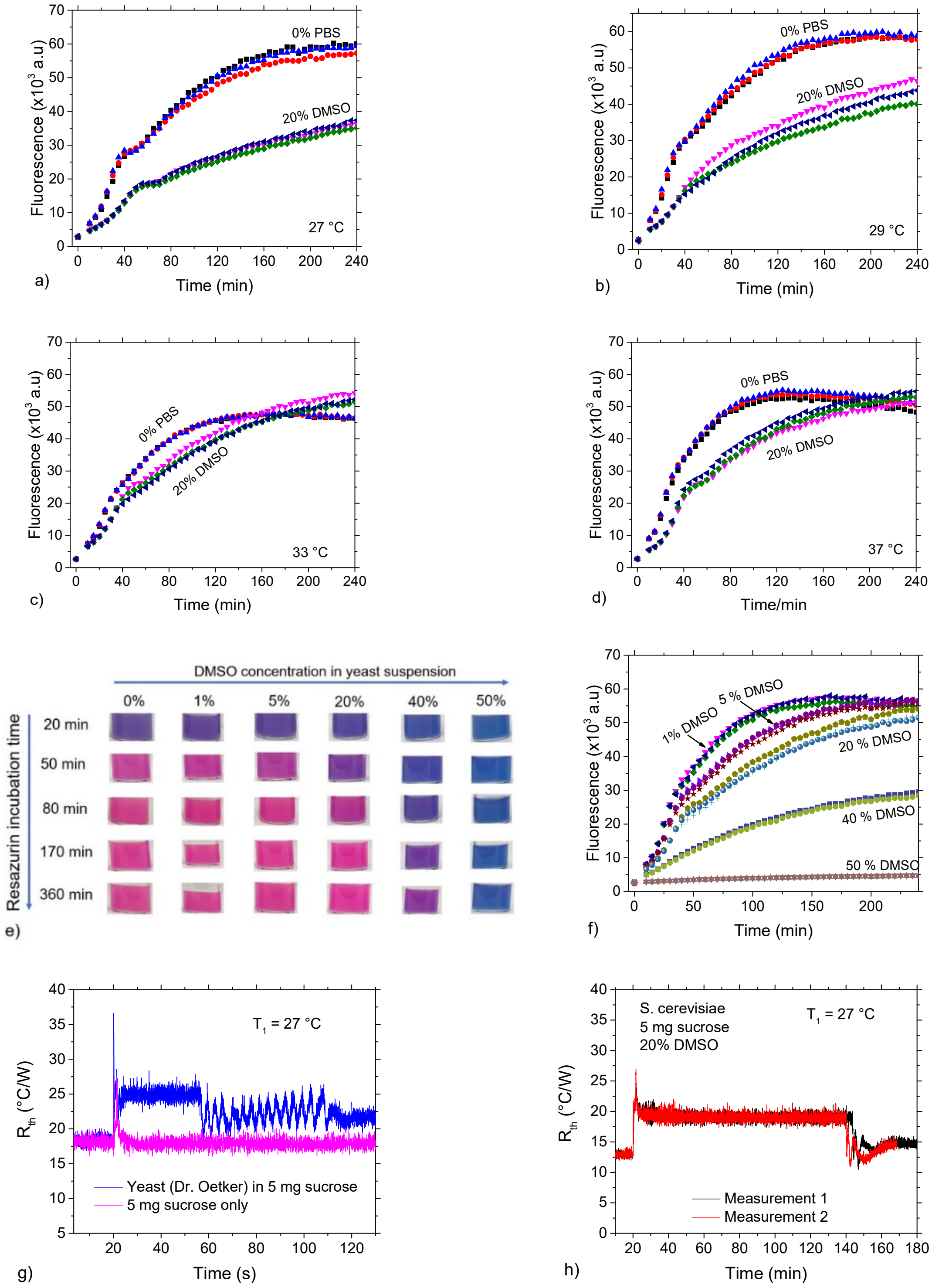

***Supplementary Figure S4. Effect of DMSO on the metabolic activity and spontaneous detachment of S. cerevisiae. a-d**, Comparison of cell metabolic activity between S. cerevisiae cells in PBS and in 20% DMSO at different temperatures showing lower cell metabolic activity for 20% in all cases. **e**, Fluorescence images displaying slower resazurin (blue) conversion to resorufin (pink) for higher DMSO concentrations. **f**, Time-dependent resorufin fluorescence intensity as a function of DMSO concentrations, showing much slower changes in intensity values with time for higher DMSO concentrations. **g**, Comparison of R_{th} as a function of time for measurements in 5 mg sucrose, with and without yeast cells. **h**, R_{th} data from two repeated measurement for yeast in 5 mg sucrose and 20% DMSO displaying delayed recovery without sustained oscillations, proofing that the oscillations are metabolic: DMSO decreases metabolic activity.*

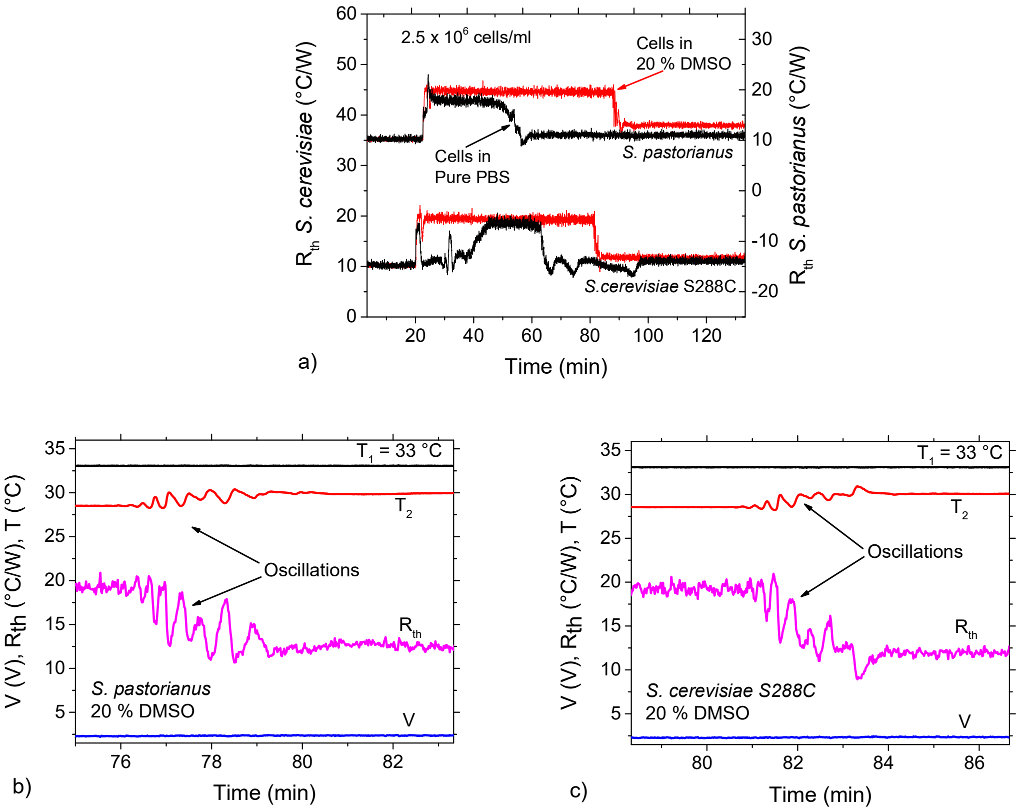

***Supplementary Figure S5. Effect of DMSO on the spontaneous detachment and oscillatory features of S. pastorianus and S. cerevisiae S288C. a**, Comparison of S. pastorianus and S. cerevisiae S288C adhesion and spontaneous detachment in pure PBS and in 20% DMSO, showing a delay in detachment for both cell types in 20% DMSO in comparison to pure PBS. In pure PBS, during initial adhesion, S. cerevisiae S288C shows a long delay before gradually attaching to the surface, which provides an extra signature for discriminating between the two cell types. Zoom in of the data for 20% DMSO-treated cells showing signal oscillations during the detachment of S. pastorianus **(b)** and S. cerevisiae S288C **(c)**. In each case, only the temperature of the liquid, T_2, and the R_{th} display oscillations, thus confirming that they are associated to events at the substrate chip-cell interface.*

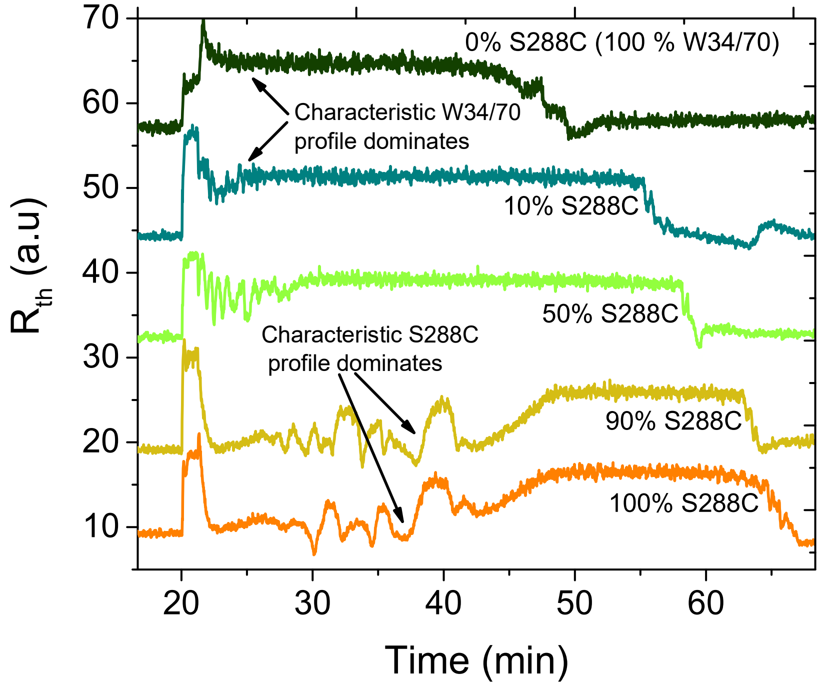

***Supplementary Figure S6. Spontaneous cell detachment analysis for cell mixtures. a**, Time-dependent R_{th} plots for different relative S. pastorianus W34/70 and S. cerevisiae S288C cell concentrations (same total concentration 2.5 × 10^6 cells/ml) displaying a short cell detachment time for a pure suspension of S. pastorianus W34/70, which increases as the relative amount of S. cerevisiae S288C is increased. The slow adsorption kinetics typical of S. cerevisiae S288C adhesion emerges as its relative amount increases: See arrows heads.*

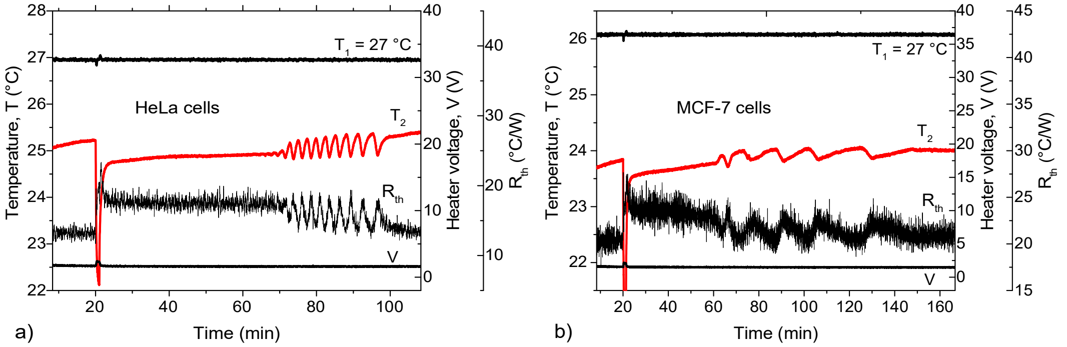

***Supplementary Figure S7. Time-dependent HeLa and MCF-7 cell oscillations. a**, HeLa cells: The temperature of the liquid, T_2, and the heat transfer resistance, R_{th}, display oscillations for 25 min while the heater voltage, V, and the set temperature T_1 (27 °C) remain constant during the entire oscillation time. **b**, Oscillations during MCF-7 cell detachment at 26 °C lasting more than 60 min.*

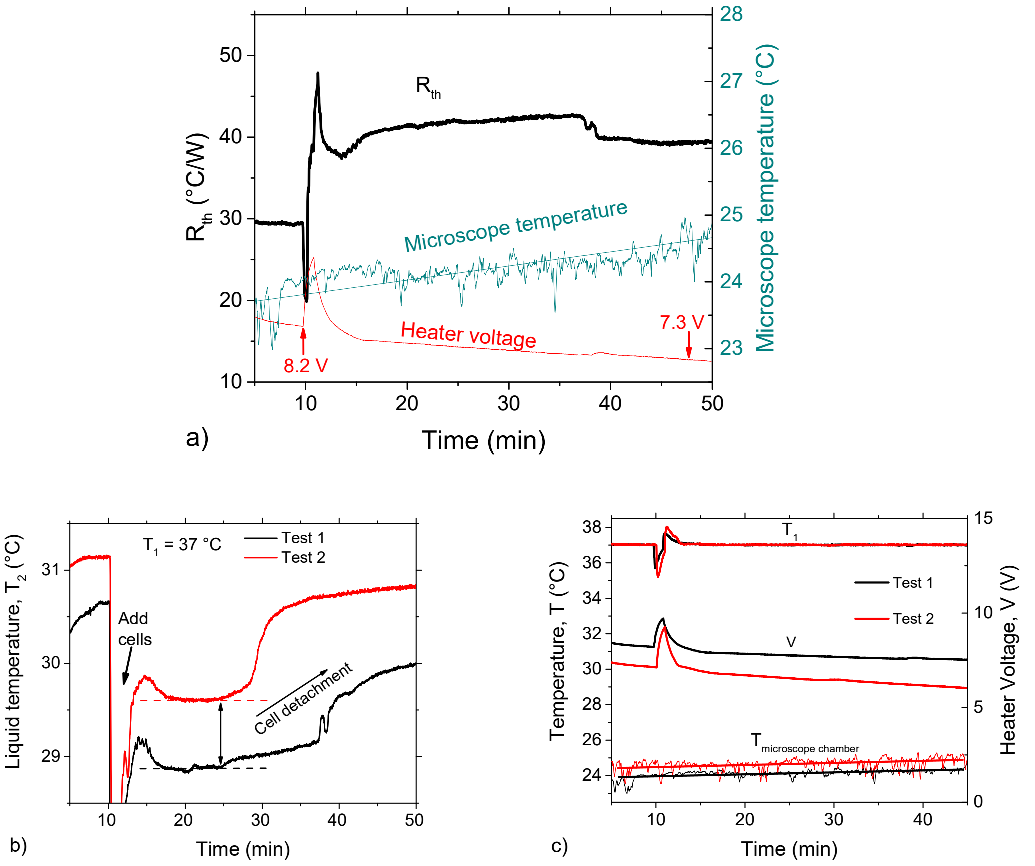

***Supplementary Figure S8. Correlative HTM measurements during live microscopy. a**, Time dependent R_{th} response for HeLa cells at 37 °C. The noisy middle signal shows changes in the temperature of the microscope chamber during measurement, while the bottom signal shows the variations in the heater voltage as a result of changes in the temperature of the chip, mostly induced by the imaging photons. **b**, Comparison of liquid temperature, T_2 signals from two HeLa cell measurements showing recoveries that being at the same time, t = 24.5 min (or 14.5 min after injection) but evolve faster for test 2 because of a higher environment temperature due to microscope warming. The average t_{d50} value is 22.5. **c**, Correlation between heater voltage and the temperature of the microscope chamber for two measurements.*

Estimation of shear rate and its effect on cell lift-off

The accuracy of the numerical result, f depends on the spacing h of the grid and the order of convergence p

$$f(h) = f(0) + a\, h^p + O(h^r)\,, \qquad r > p$$

Where *f(0)* is the 'exact' value and the coefficient a is independent of the grid spacing *h*.
The error *E* is given by the difference between the *f(0)* and the approximate solution *f(h)*$E = f(h) - f(0) = a\, h^p + O(h^r)\,,\ r > p$

If for a chosen value of *h*, the solutions *f(h)* and *f(h/l)* for a positive (integral) grid refinement ratio l are computed, the equation can be solved for *a* and *f(0)*. This yields values *p*th order accurate. Since this approximation can again be described with the same equation, repeating the process results in a value for *f(0)* that is even more accurate. Extrapolating the value of *f(0)* from *f(h)* and *f(h)* according to this approach is called Richardson extrapolation ([1] P. J. Roache. Fundamentals of Computational Fluid Dynamics. Hermosa Publishers,1998.).

f is defined as the average shear rate on the bottom surface of the fluid cell. We computed the solutions *f1*, *f2* and *f3* for a 800 µm, 400 µm and 200 µm grid respectively. This corresponds to a grid refinement ration l of 2. The result is displayed in table 1.

**Shear rate (1/s)**	***f1*: 800 µm**	***f2*: 400 µm**	***f3*: 200 µm**
**37°C**	1.3654	1.3488	1.3421

The order of convergence amounts:

$$p = \frac{\ln(\frac{f_1 - f_2}{f_2 - f_3})}{\ln(l)} = 1.30895$$

Applying Richardson extrapolation using the two finest grids:

$$f(0) \approx f_3 + \frac{f_3 - f_2}{l^p - 1} = 1.33757\ s^{-1}$$

The grid convergence index (GCI) amounts then

$$GCI_{12} = 2.49\%, GCI_{23} = 1.01\%$$

The solutions lie within the asymptotic range of convergence as

$$\frac{GCI_{12}}{l^p GCI_{23}} = 0.995$$

The average shear rate at the sensor surface thus amounts (1.337 ± 0.014) s^{-1}.

According to Krishnan *et al.* (reference [73] in the main manuscript), the onset of lifting depends on the relation Reynolds numbers Re^2/Re_g , with $Re = \dot{\gamma}a^2/\nu$ and $Re_g = U_g a/\nu$. Re is the Reynold number, which is given by the ratio between the inertia of a particle with radius a and the drag force due to the viscosity of the solvent ν. In this case the particle has a velocity due to the local shear rate $\dot{\gamma}$. $Re_g$the velocity is given by the sedimentation velocity $U_g = \frac{2a^2\Delta\rho g}{9\nu}$ of a (spherical) particle.

Therefore,

$$\frac{Re^2}{Re_g} = \frac{(\dot{\gamma}a^2/\nu)^2}{\frac{2a^2\Delta\rho}{9\nu}a/\nu} = \frac{9\dot{\gamma}^2 a}{2\Delta\rho g}.$$

The onset of lifting is around $\frac{Re^2}{Re_g} = 10$ according to ref. [73]. Assuming that for a cell $\Delta\rho = 0.1\ kg/l$ and $a = 10^{-5}\ m$, we required that $\dot{\gamma} > 500\ s^{-1}$ to obtain convection-driven lifting, which is certainly not the case here.
